# Supplementary material for: Pediatric Emergency Medicine Didactics and Simulation (PEMDAS): Pediatric Sedation Complications
Source: MedEdPORTAL. 2024 Feb 13;20:11384. doi: 10.15766/mep_2374-8265.11384 (PMC10861802; doi:10.15766/mep_2374-8265.11384)
Supplement: Supplementary file 1 — Sedation Simulation Cases.docxSedation Simulation Patients.docxCritical Actions Checklist.docxSedation Simulation Equipment.docxSedation Simulation X-Ray Images.docxSedation Simulation Debriefing Materials.docxSedation Simulation Evaluation.docxPropofol and Ketamine.pptx [file mep_2374-8265.11384-s001.zip › C. Critical Actions Checklist.docx]

**Appendix C: Critical Actions Checklist**

Patient #1 (Adam):

- Obtain verbal consent from the guardian/parent of the patient
  - Explain sedation plan
  - Discuss risks, benefits and alternatives of sedation
- Determine if patient is appropriate for ED sedation
  - Obtain a pre-sedation history
  - Perform a pre-sedation physical exam
  - Assign an ASA classification
- Prepare the room and patient appropriately for the sedation
  - Obtain vascular access
  - Set up emergency airway equipment including appropriately sized BVM
  - Set up appropriate patient monitoring (capnography, suction, pulse oximetry, cardiac monitoring)
- Perform a pre-sedation “time out” with RN, guardian and procedure performing physician present
- Administer correct doses of propofol
- Recognize and appropriately manage apnea with airway repositioning and BVM ventilation
- Recognize and appropriately manage hypotension with a 20 cc/kg NS bolus

Patient #2 (Sam):

- Assign ASA classifications and determine which patient is appropriate for ED sedation
- Obtain verbal consent from the guardian/parent of the patient
  - Explain sedation plan
  - Discuss risks, benefits and alternatives of sedation
- Confirm the patient is appropriate for ED sedation
  - Obtain a pre-sedation history
  - Perform a pre-sedation physical exam
  - Assign an ASA classification
- Confirm the room and patient are ready for sedation
  - Double check equipment is available including appropriately sized BVM, pulse oximeter, capnography, cardiac monitoring, and suction
- Perform a pre-sedation “time out” with RN, guardian and procedure performing physician present
- Administer correct doses of Ketamine
- Recognize and appropriately manage laryngospasm with:
  - Vigorous airway repositioning with BVM
  - Laryngospasm notch pressure with jaw thrust (Larson’s maneuver)
  - Administration of succinylcholine 1 mg/kg IV
  - Continued BVM ventilation until the patient has spontaneous respiratory effort and is maintaining airway and saturations
