# Supplementary material for: Pediatric Emergency Medicine Didactics and Simulation (PEMDAS): Pediatric Sedation Complications
Source: MedEdPORTAL. 2024 Feb 13;20:11384. doi: 10.15766/mep_2374-8265.11384 (PMC10861802; doi:10.15766/mep_2374-8265.11384)
Supplement: Supplementary file 1 — Sedation Simulation Cases.docxSedation Simulation Patients.docxCritical Actions Checklist.docxSedation Simulation Equipment.docxSedation Simulation X-Ray Images.docxSedation Simulation Debriefing Materials.docxSedation Simulation Evaluation.docxPropofol and Ketamine.pptx [file mep_2374-8265.11384-s001.zip › H. Propofol and Ketamine.pptx]

## Slide 1
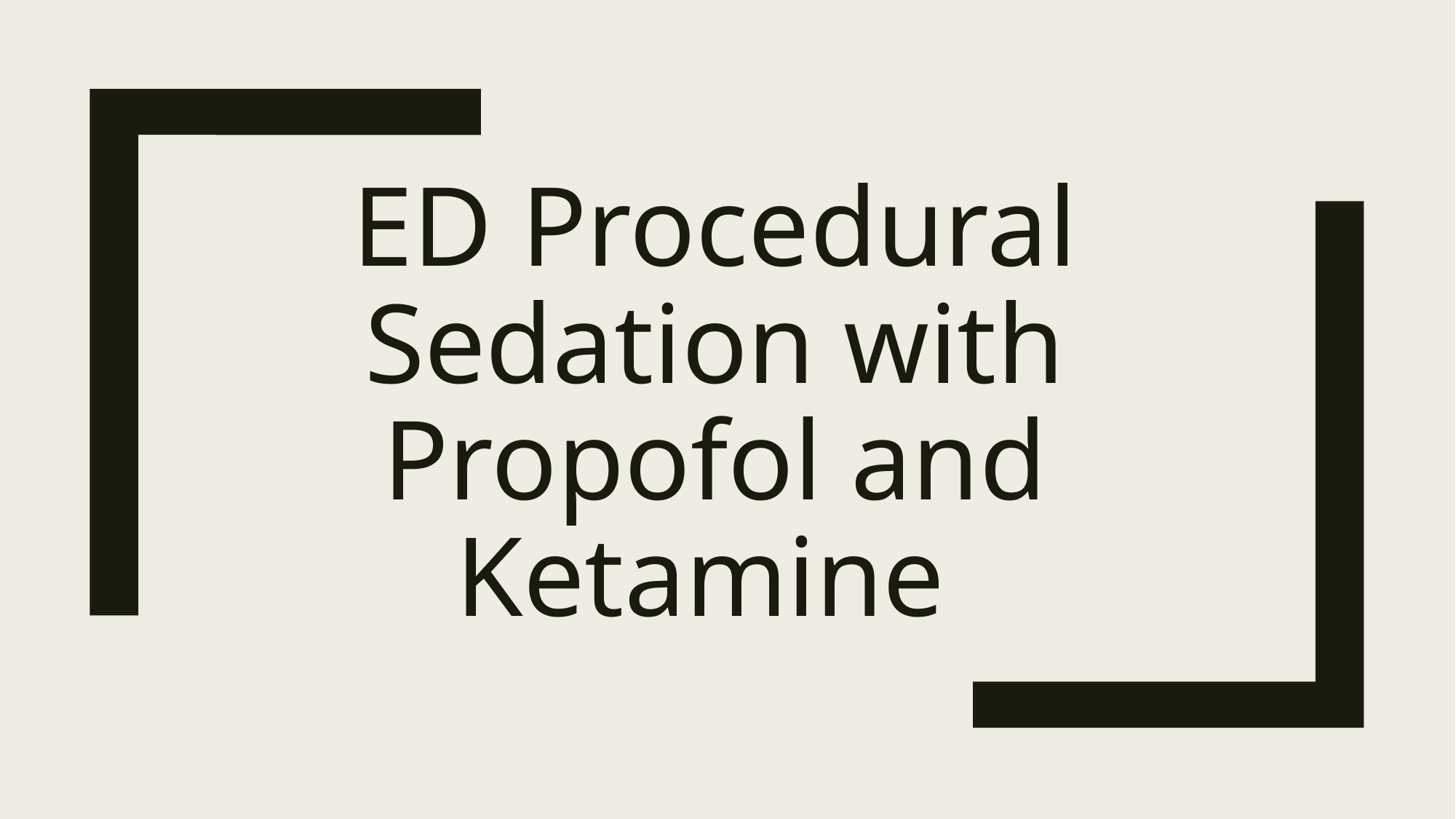

# ED Procedural Sedation with Propofol and Ketamine

## Slide 2
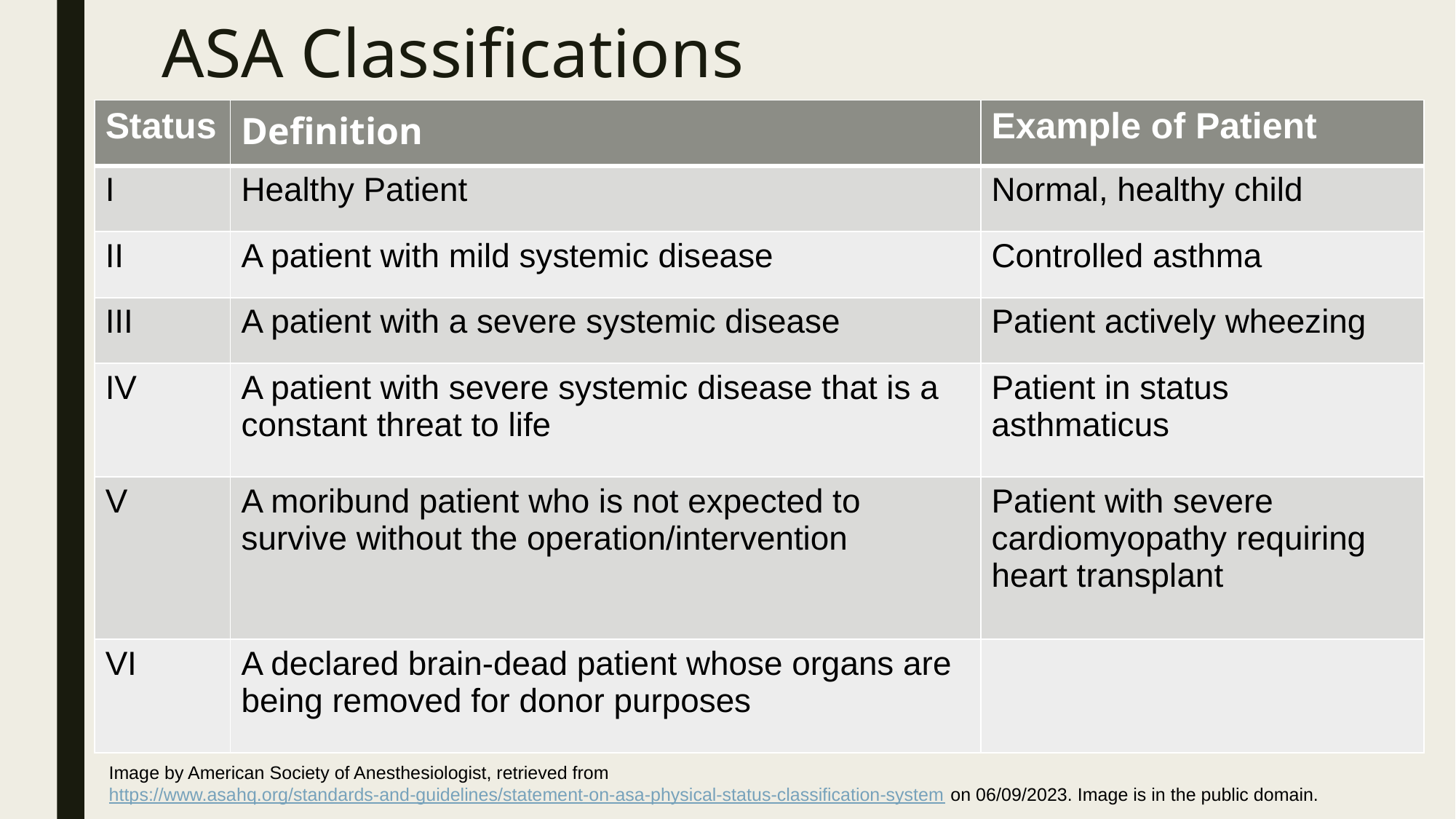

# ASA Classifications
| Status | Definition | Example of Patient |
| --- | --- | --- |
| I | Healthy Patient | Normal, healthy child |
| II | A patient with mild systemic disease | Controlled asthma |
| III | A patient with a severe systemic disease | Patient actively wheezing |
| IV | A patient with severe systemic disease that is a constant threat to life | Patient in status asthmaticus |
| V | A moribund patient who is not expected to survive without the operation/intervention | Patient with severe cardiomyopathy requiring heart transplant |
| VI | A declared brain-dead patient whose organs are being removed for donor purposes | |
Image by American Society of Anesthesiologist, retrieved from https://www.asahq.org/standards-and-guidelines/statement-on-asa-physical-status-classification-system on 06/09/2023. Image is in the public domain.

## Slide 3
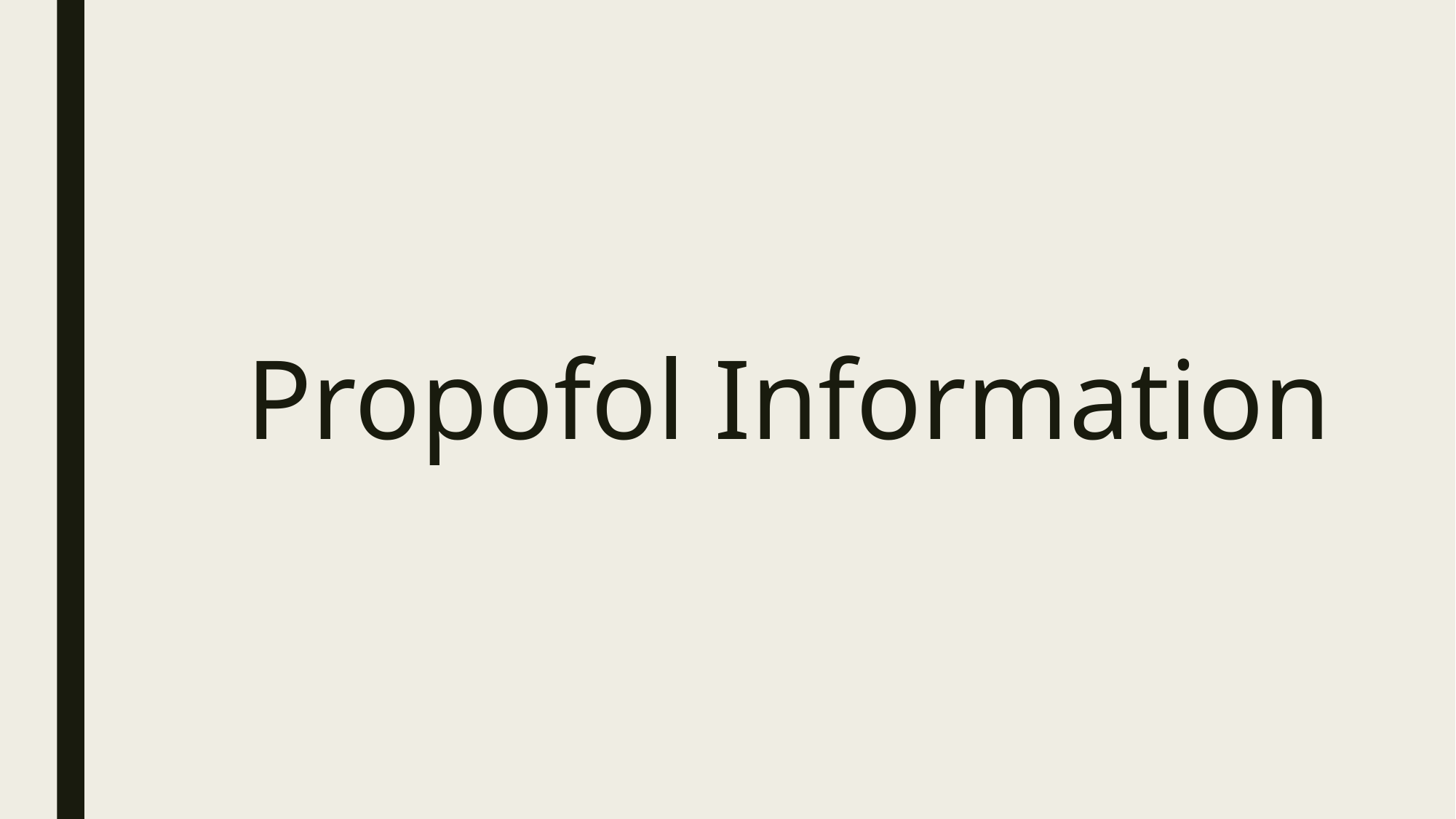

# Propofol Information

## Slide 4
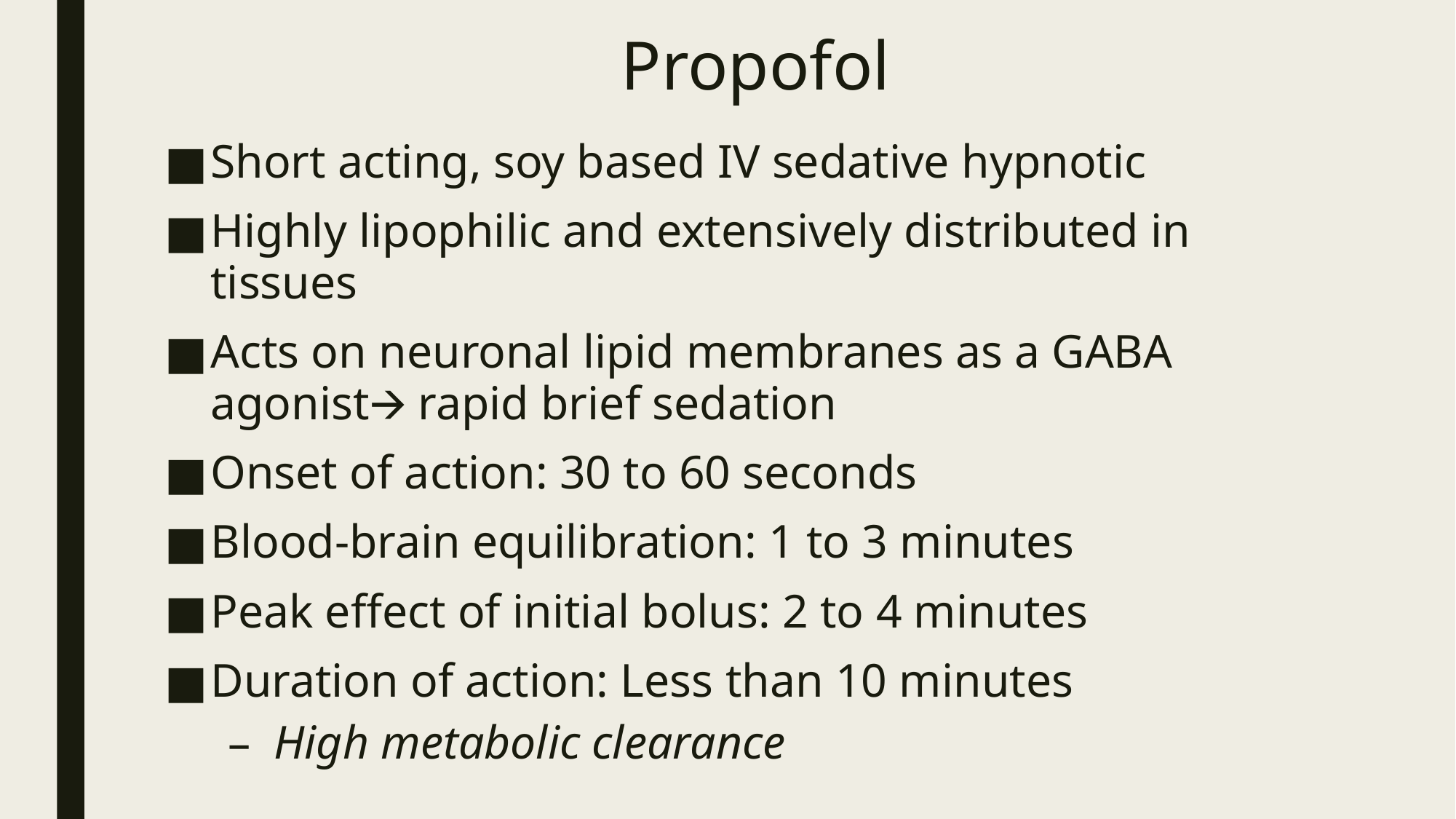

# Propofol
Short acting, soy based IV sedative hypnotic
Highly lipophilic and extensively distributed in tissues
Acts on neuronal lipid membranes as a GABA agonist🡪 rapid brief sedation
Onset of action: 30 to 60 seconds
Blood-brain equilibration: 1 to 3 minutes
Peak effect of initial bolus: 2 to 4 minutes
Duration of action: Less than 10 minutes
High metabolic clearance

## Slide 5
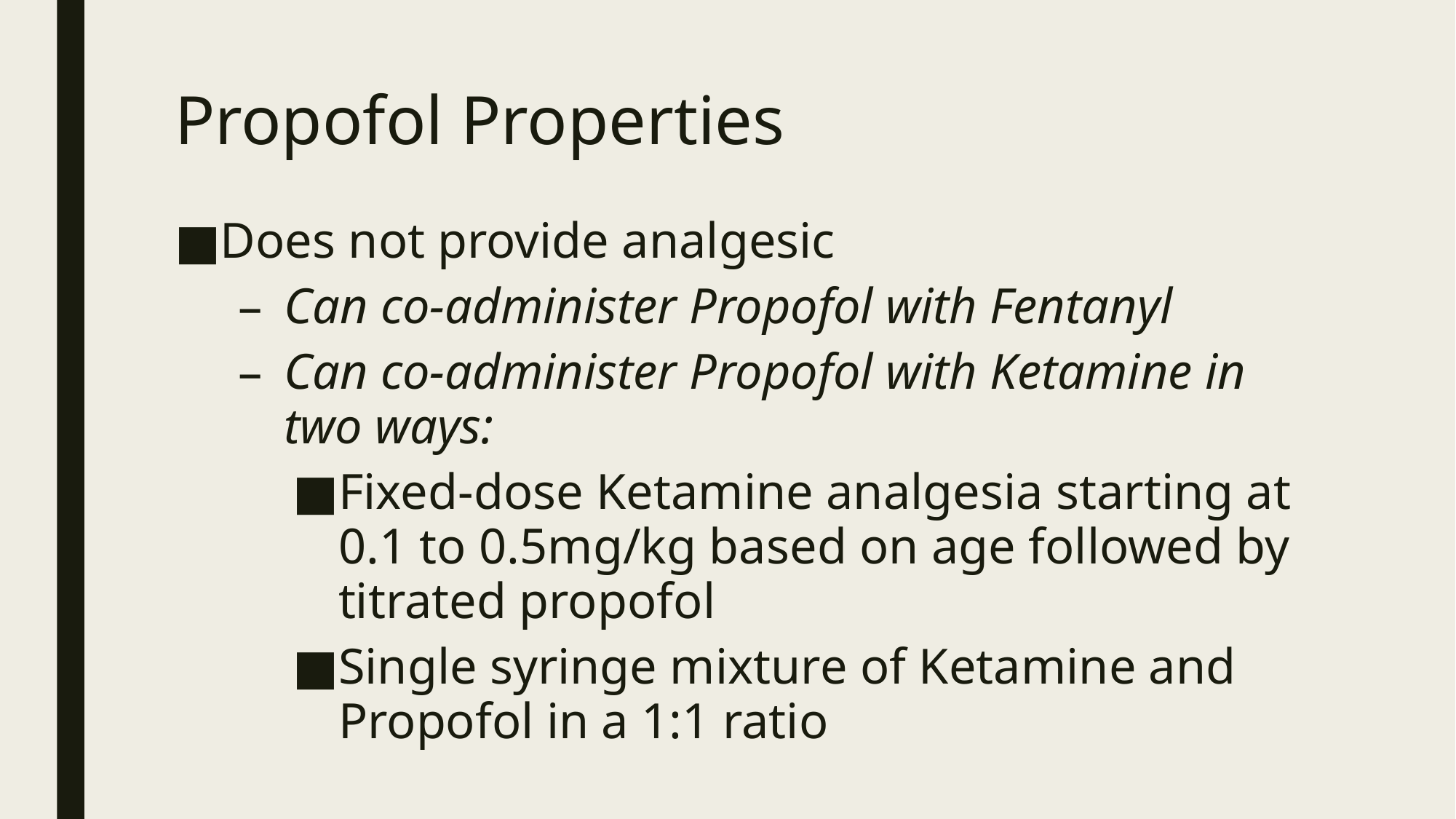

# Propofol Properties
Does not provide analgesic
Can co-administer Propofol with Fentanyl
Can co-administer Propofol with Ketamine in two ways:
Fixed-dose Ketamine analgesia starting at 0.1 to 0.5mg/kg based on age followed by titrated propofol
Single syringe mixture of Ketamine and Propofol in a 1:1 ratio

## Slide 6
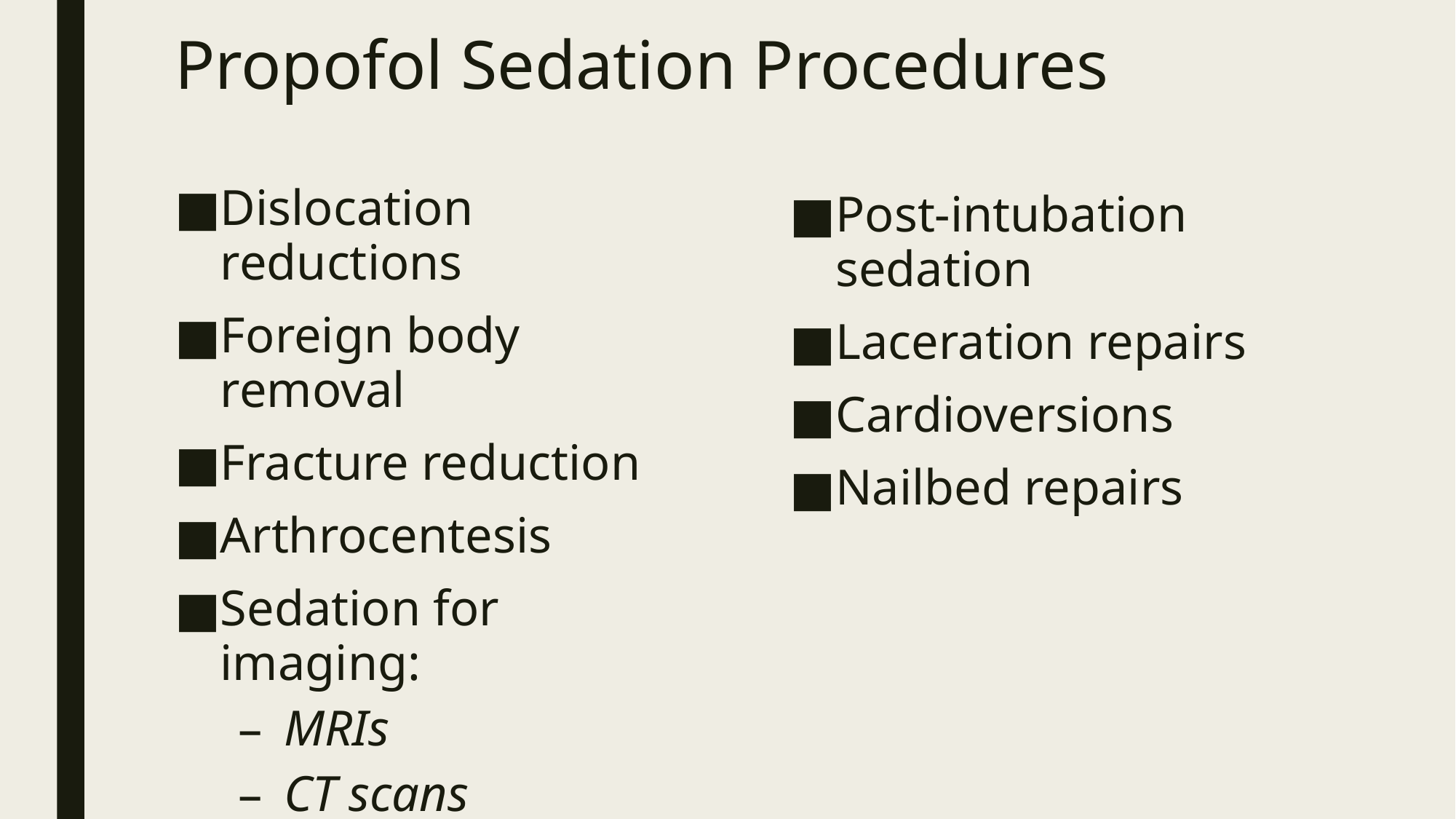

# Propofol Sedation Procedures
Dislocation reductions
Foreign body removal
Fracture reduction
Arthrocentesis
Sedation for imaging:
MRIs
CT scans
Post-intubation sedation
Laceration repairs
Cardioversions
Nailbed repairs

## Slide 7
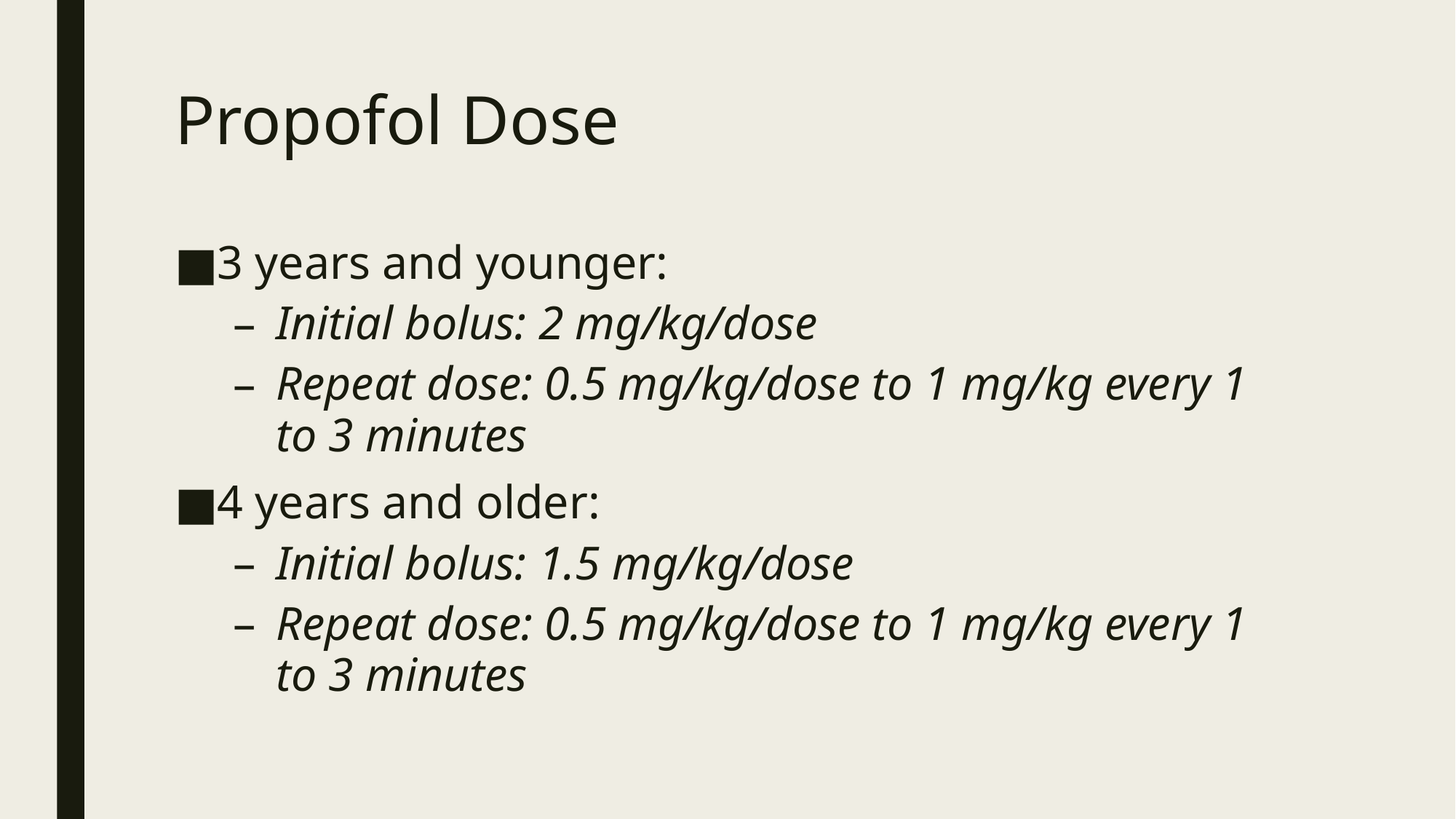

# Propofol Dose
3 years and younger:
Initial bolus: 2 mg/kg/dose
Repeat dose: 0.5 mg/kg/dose to 1 mg/kg every 1 to 3 minutes
4 years and older:
Initial bolus: 1.5 mg/kg/dose
Repeat dose: 0.5 mg/kg/dose to 1 mg/kg every 1 to 3 minutes

## Slide 8
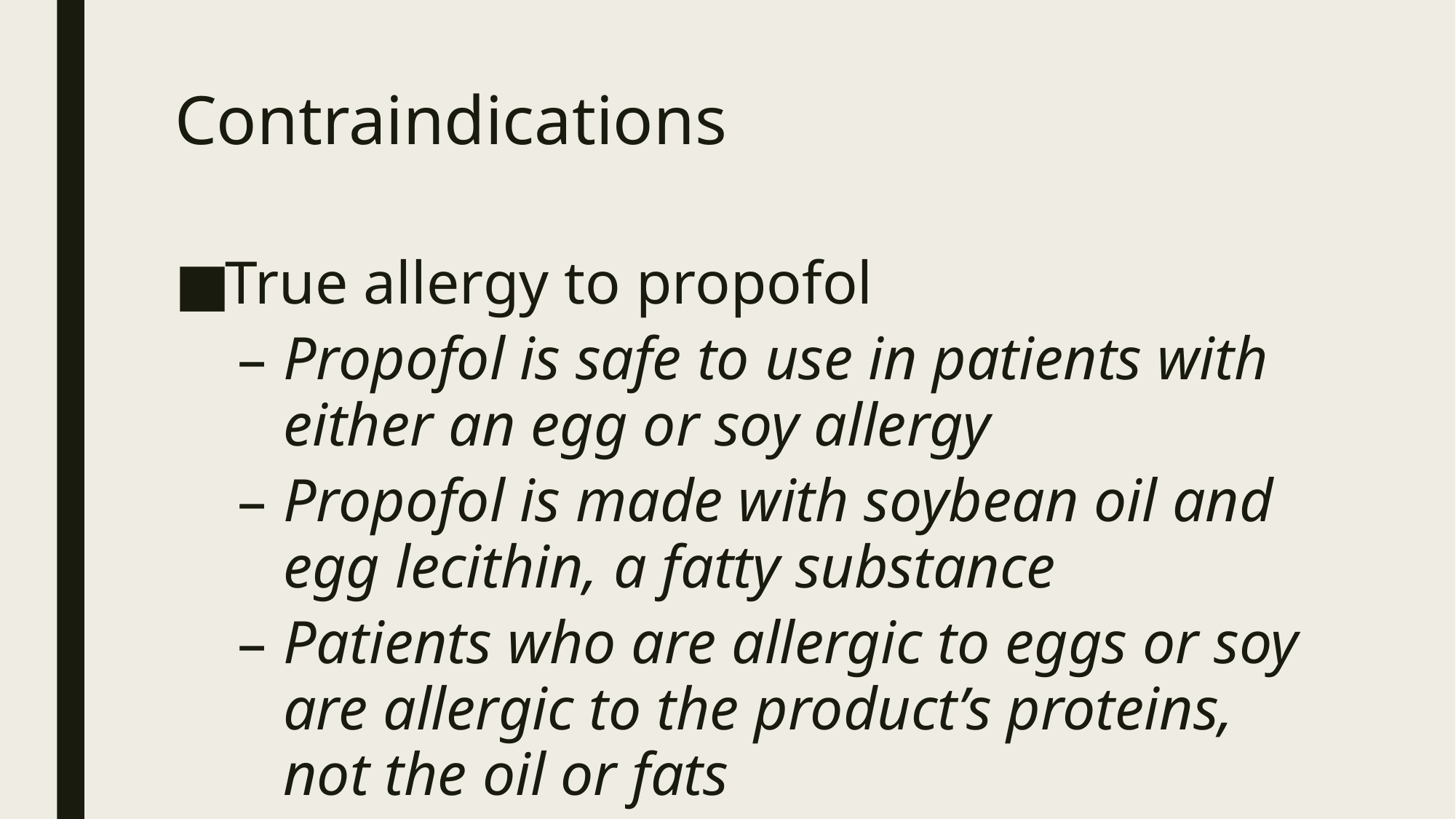

# Contraindications
True allergy to propofol
Propofol is safe to use in patients with either an egg or soy allergy
Propofol is made with soybean oil and egg lecithin, a fatty substance
Patients who are allergic to eggs or soy are allergic to the product’s proteins, not the oil or fats

## Slide 9
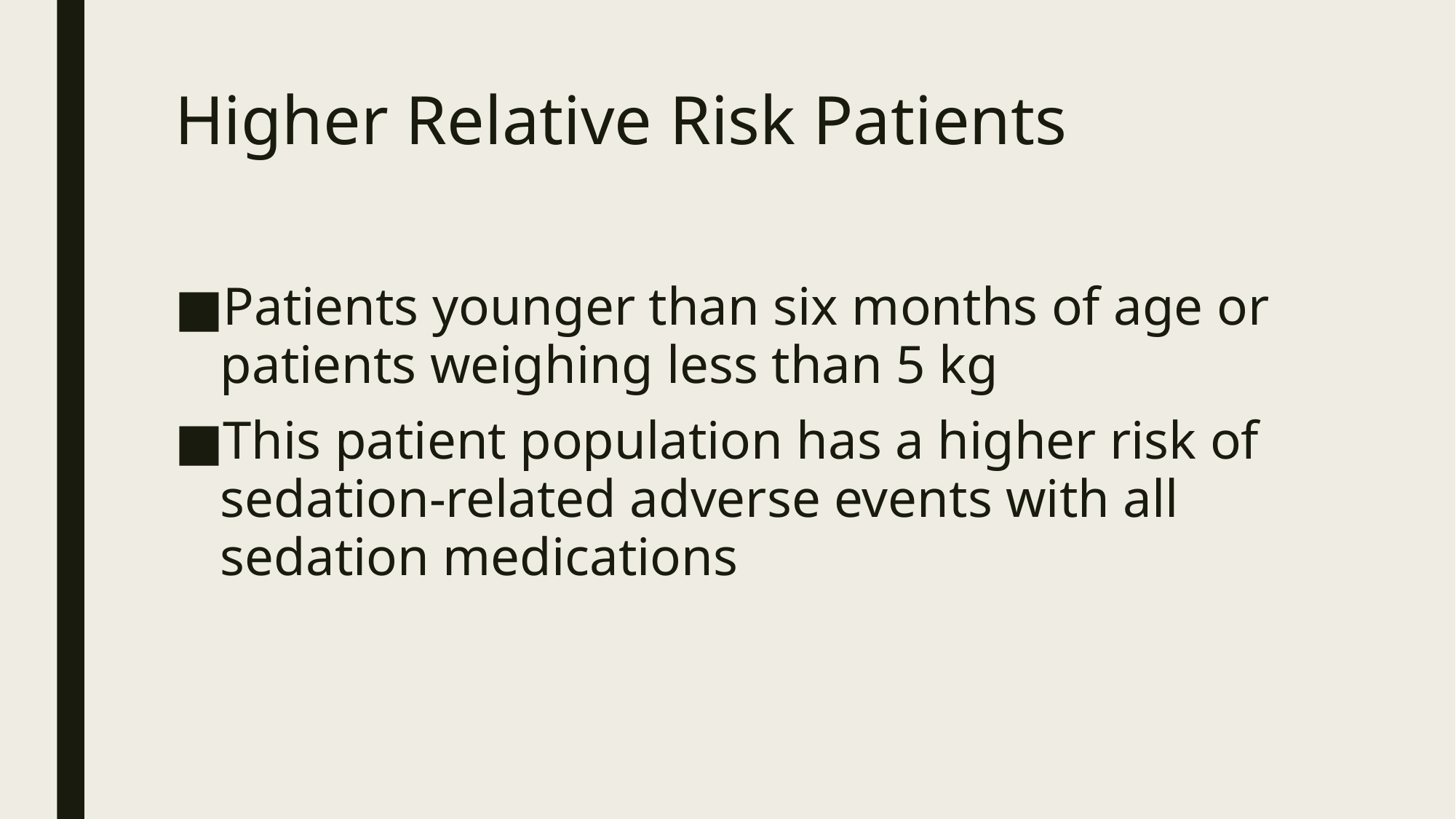

# Higher Relative Risk Patients
Patients younger than six months of age or patients weighing less than 5 kg
This patient population has a higher risk of sedation-related adverse events with all sedation medications

## Slide 10
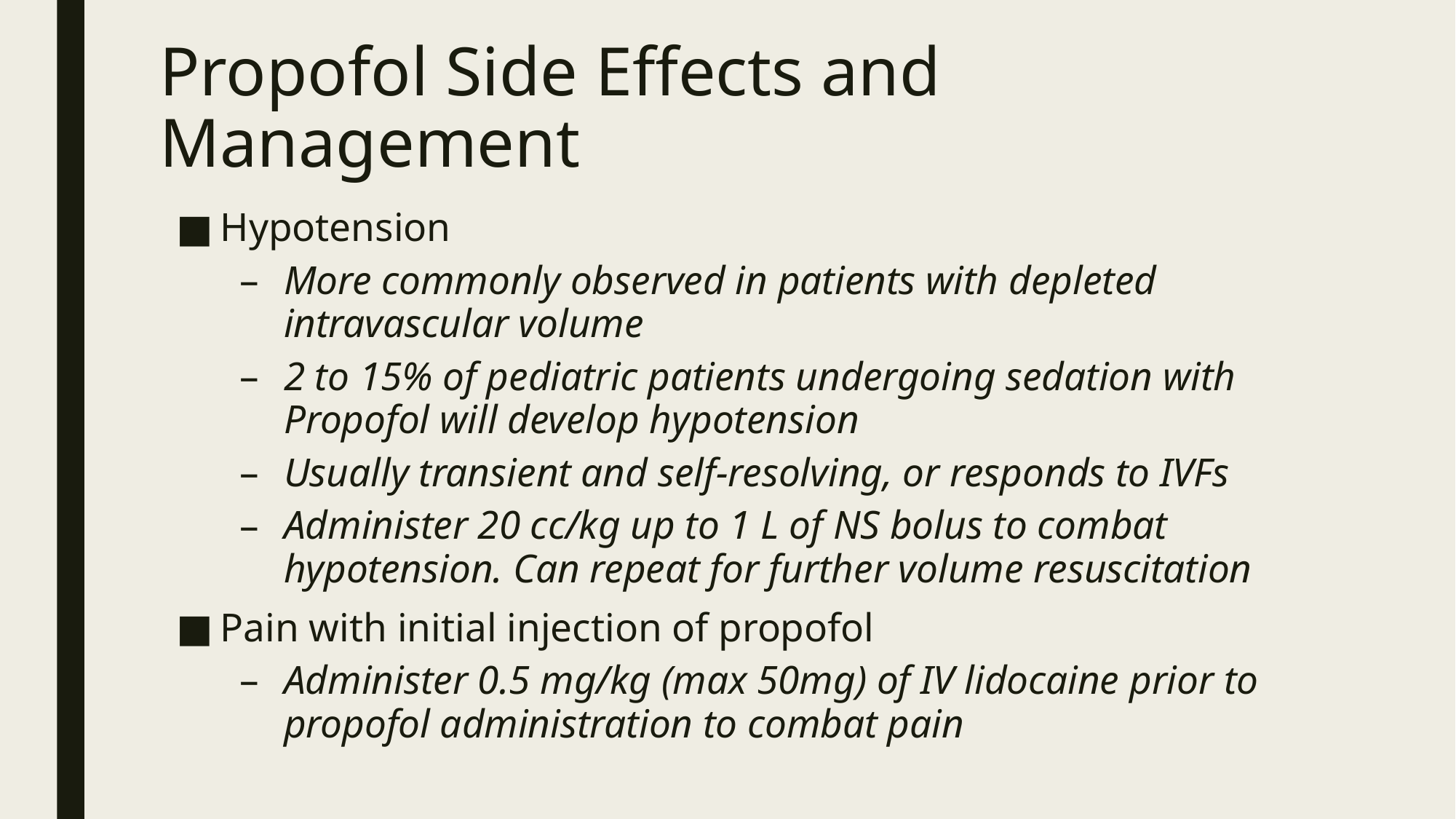

# Propofol Side Effects and Management
Hypotension
More commonly observed in patients with depleted intravascular volume
2 to 15% of pediatric patients undergoing sedation with Propofol will develop hypotension
Usually transient and self-resolving, or responds to IVFs
Administer 20 cc/kg up to 1 L of NS bolus to combat hypotension. Can repeat for further volume resuscitation
Pain with initial injection of propofol
Administer 0.5 mg/kg (max 50mg) of IV lidocaine prior to propofol administration to combat pain

## Slide 11
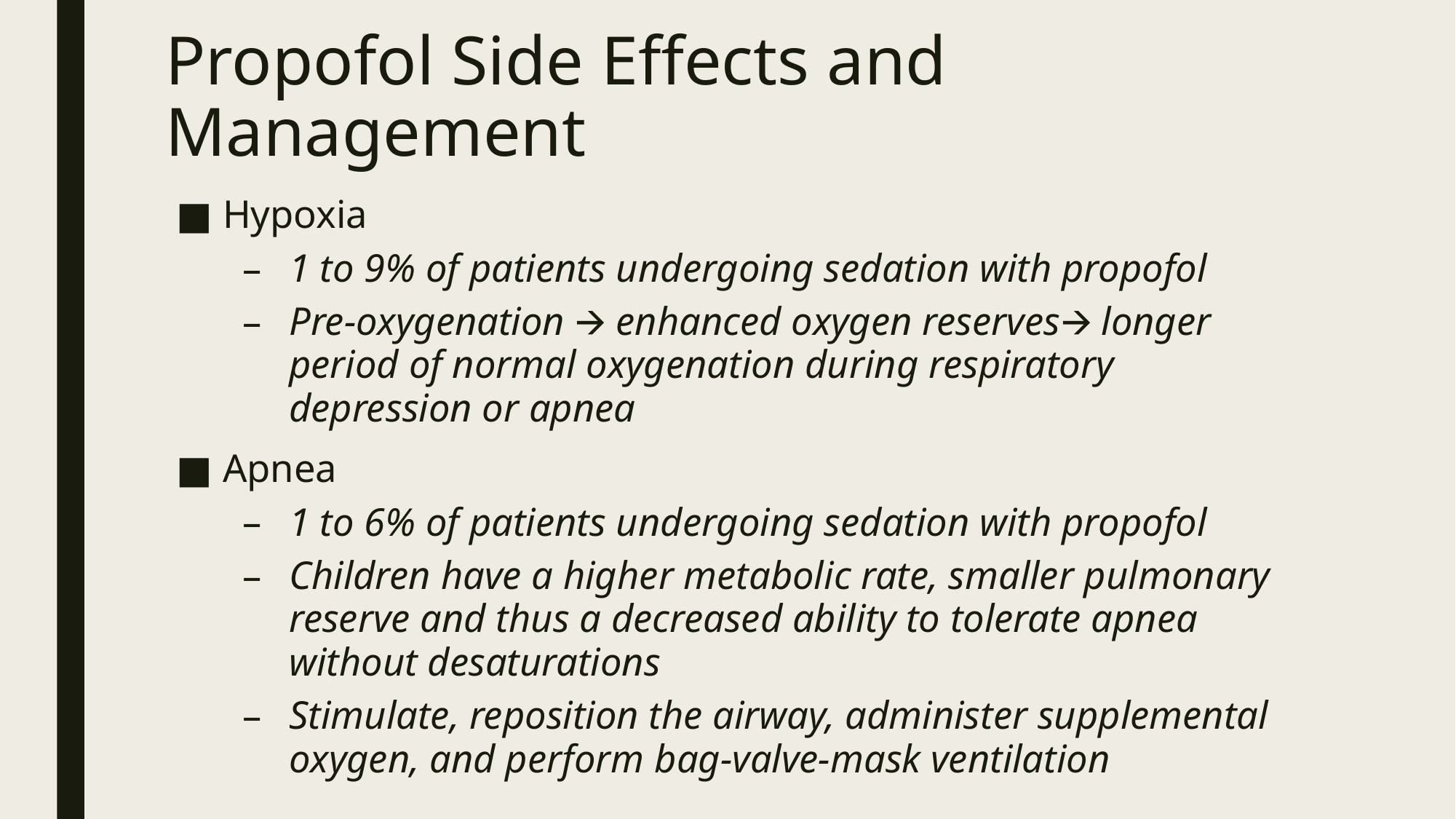

# Propofol Side Effects and Management
Hypoxia
1 to 9% of patients undergoing sedation with propofol
Pre-oxygenation 🡪 enhanced oxygen reserves🡪 longer period of normal oxygenation during respiratory depression or apnea
Apnea
1 to 6% of patients undergoing sedation with propofol
Children have a higher metabolic rate, smaller pulmonary reserve and thus a decreased ability to tolerate apnea without desaturations
Stimulate, reposition the airway, administer supplemental oxygen, and perform bag-valve-mask ventilation

## Slide 12
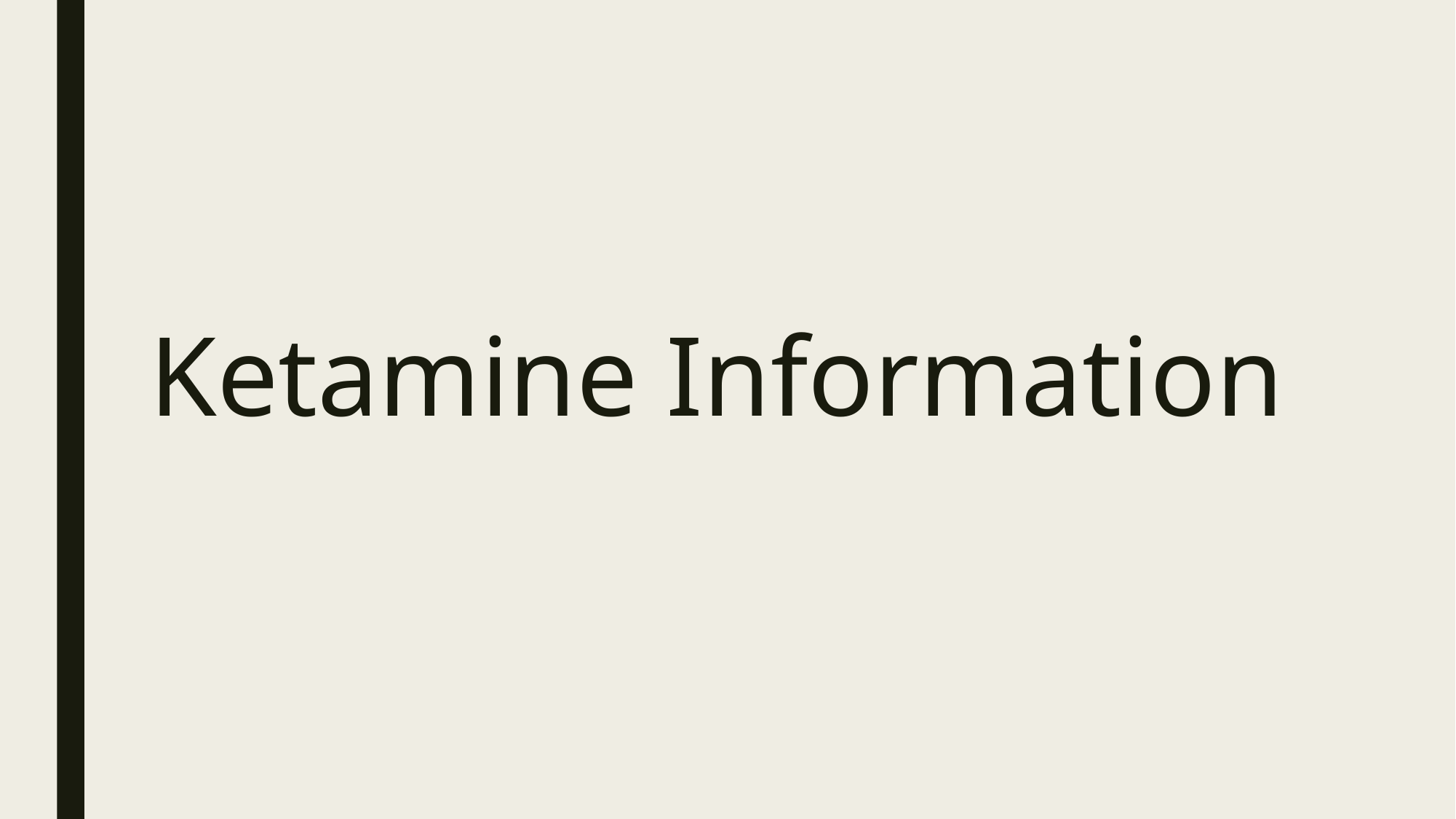

# Ketamine Information

## Slide 13
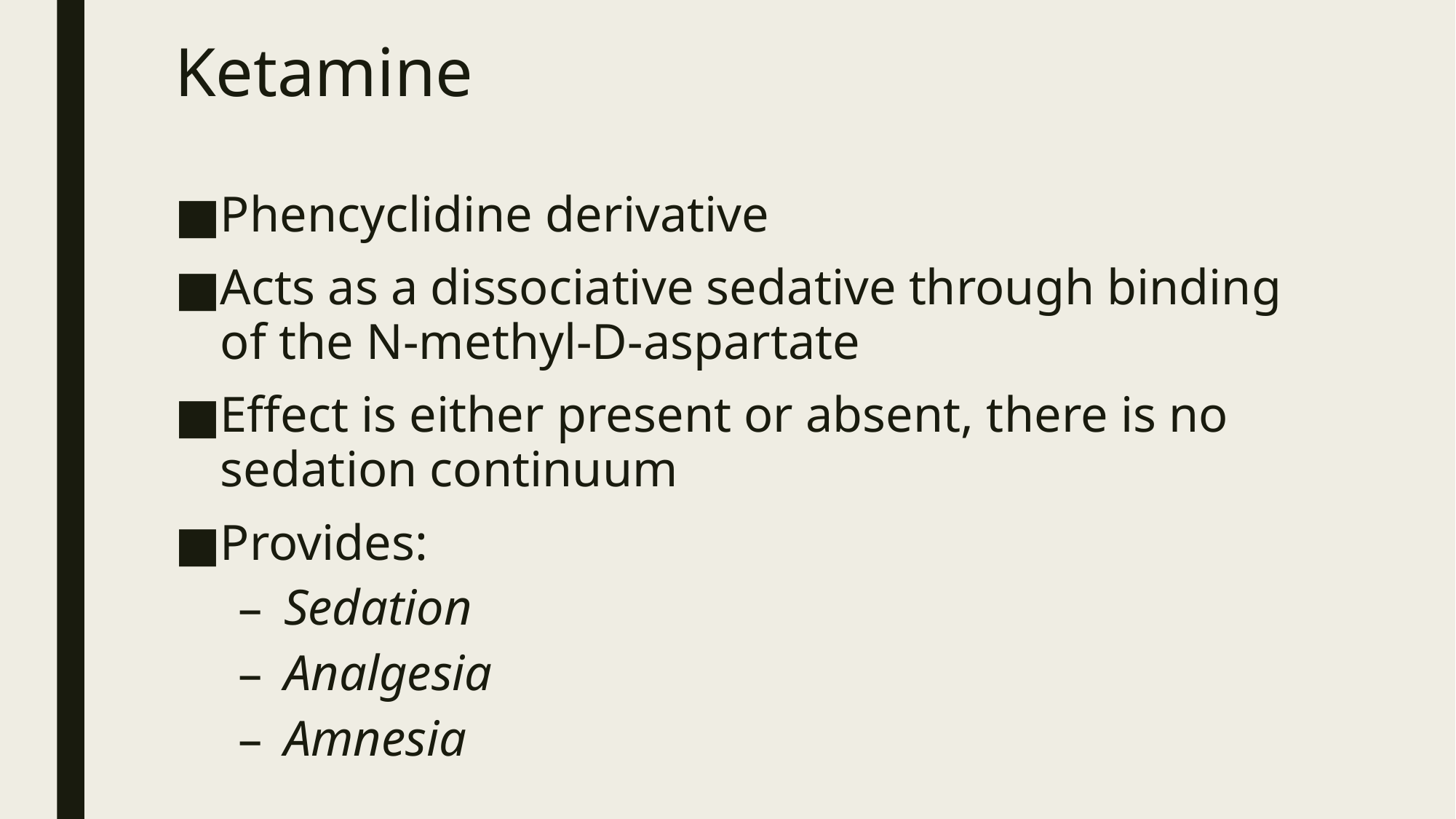

# Ketamine
Phencyclidine derivative
Acts as a dissociative sedative through binding of the N-methyl-D-aspartate
Effect is either present or absent, there is no sedation continuum
Provides:
Sedation
Analgesia
Amnesia

## Slide 14
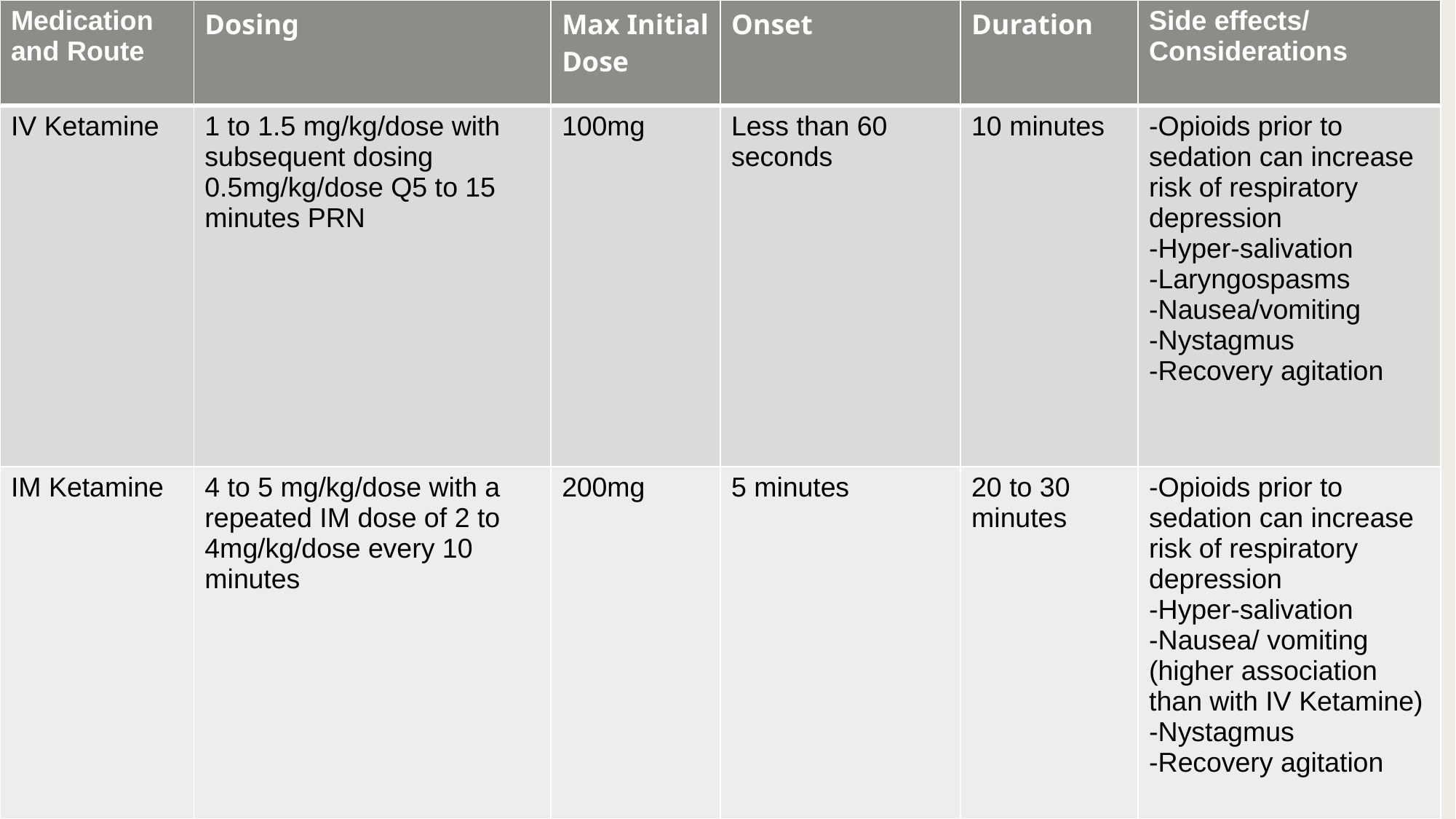

| Medication and Route | Dosing | Max Initial Dose | Onset | Duration | Side effects/ Considerations |
| --- | --- | --- | --- | --- | --- |
| IV Ketamine | 1 to 1.5 mg/kg/dose with subsequent dosing 0.5mg/kg/dose Q5 to 15 minutes PRN | 100mg | Less than 60 seconds | 10 minutes | -Opioids prior to sedation can increase risk of respiratory depression -Hyper-salivation -Laryngospasms -Nausea/vomiting -Nystagmus -Recovery agitation |
| IM Ketamine | 4 to 5 mg/kg/dose with a repeated IM dose of 2 to 4mg/kg/dose every 10 minutes | 200mg | 5 minutes | 20 to 30 minutes | -Opioids prior to sedation can increase risk of respiratory depression -Hyper-salivation -Nausea/ vomiting (higher association than with IV Ketamine) -Nystagmus -Recovery agitation |

## Slide 15
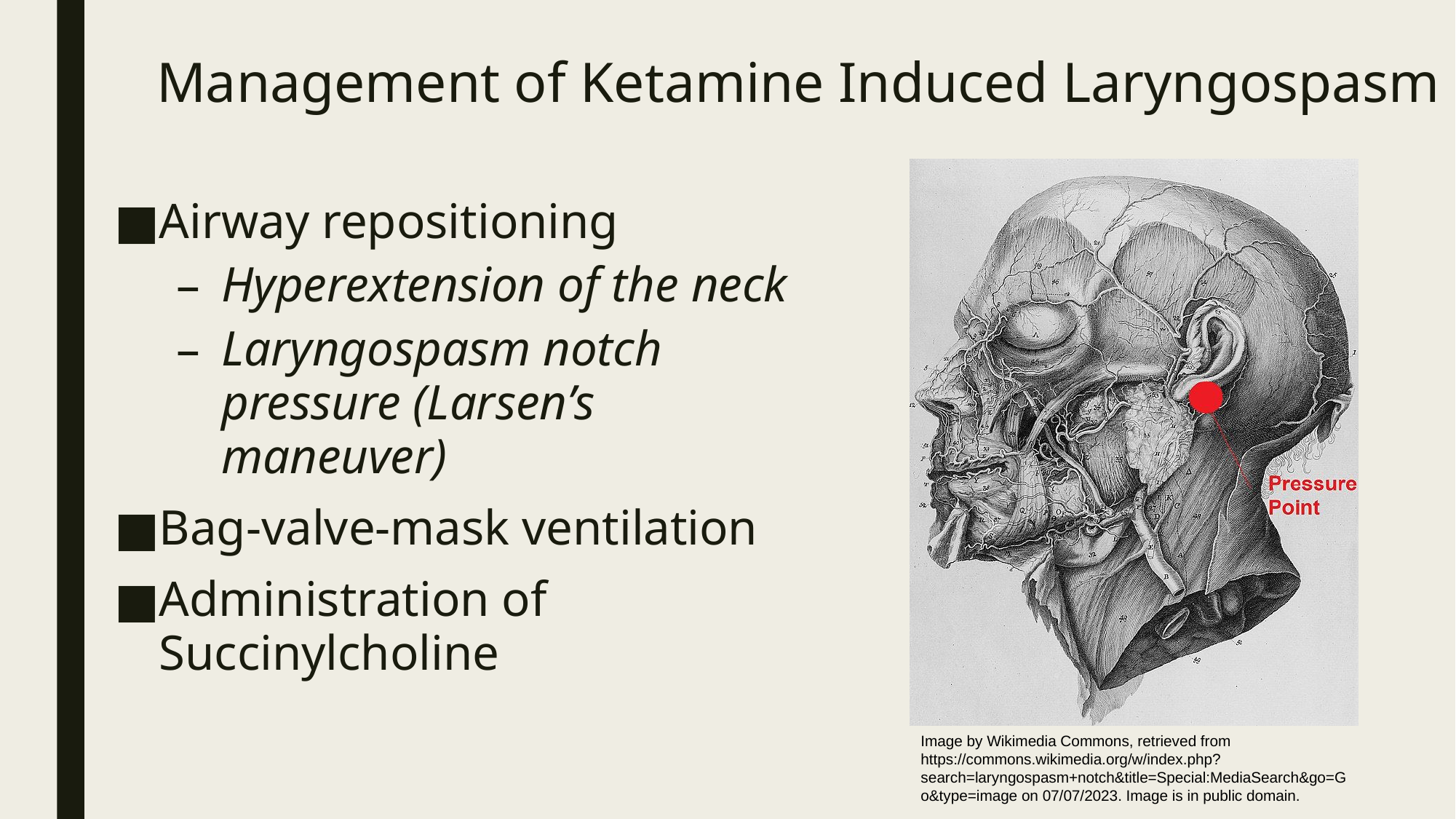

# Management of Ketamine Induced Laryngospasm
Airway repositioning
Hyperextension of the neck
Laryngospasm notch pressure (Larsen’s maneuver)
Bag-valve-mask ventilation
Administration of Succinylcholine
Image by Wikimedia Commons, retrieved from  https://commons.wikimedia.org/w/index.php?search=laryngospasm+notch&title=Special:MediaSearch&go=Go&type=image on 07/07/2023. Image is in public domain.

## Slide 16
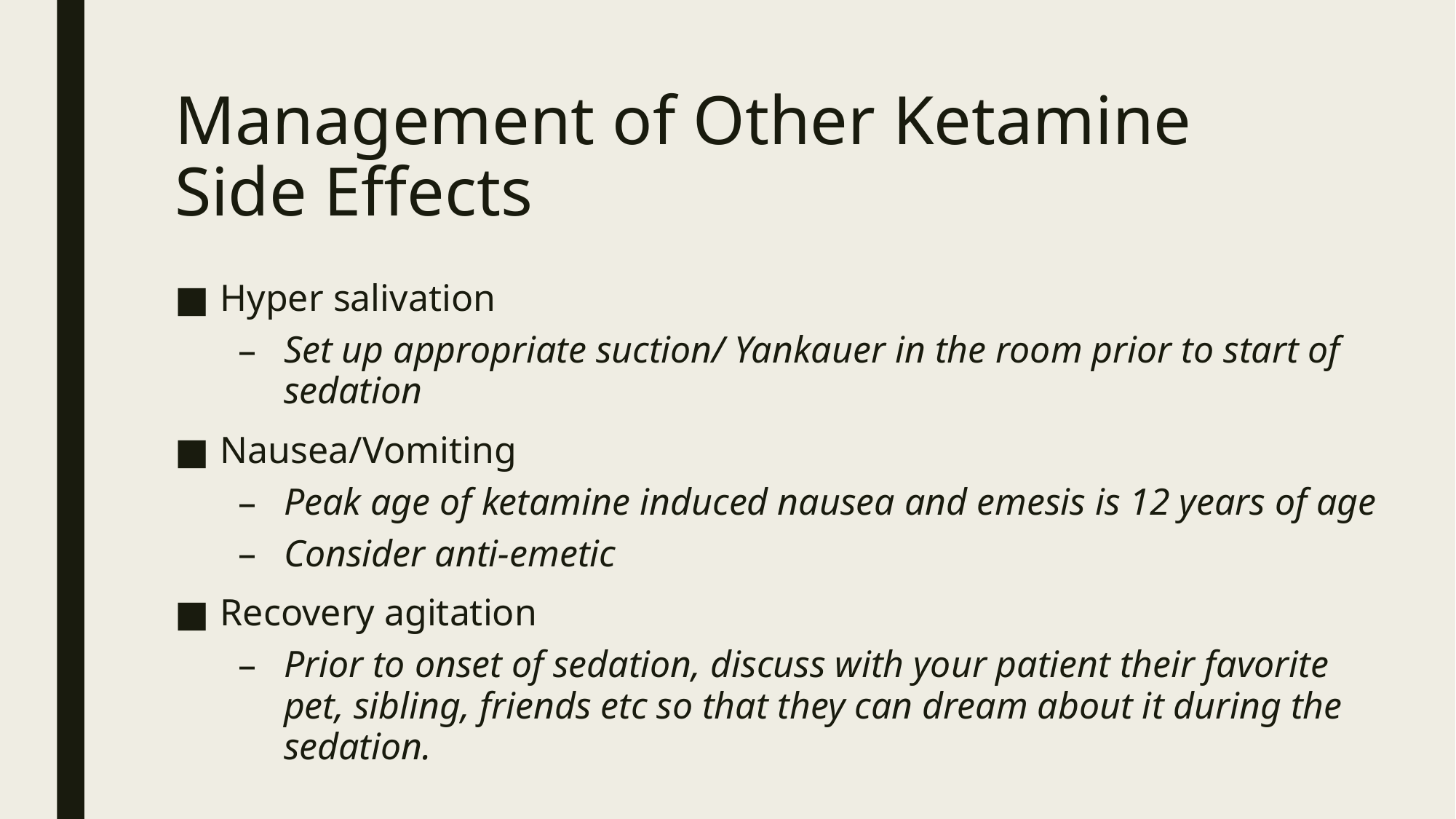

# Management of Other Ketamine Side Effects
Hyper salivation
Set up appropriate suction/ Yankauer in the room prior to start of sedation
Nausea/Vomiting
Peak age of ketamine induced nausea and emesis is 12 years of age
Consider anti-emetic
Recovery agitation
Prior to onset of sedation, discuss with your patient their favorite pet, sibling, friends etc so that they can dream about it during the sedation.

## Slide 17
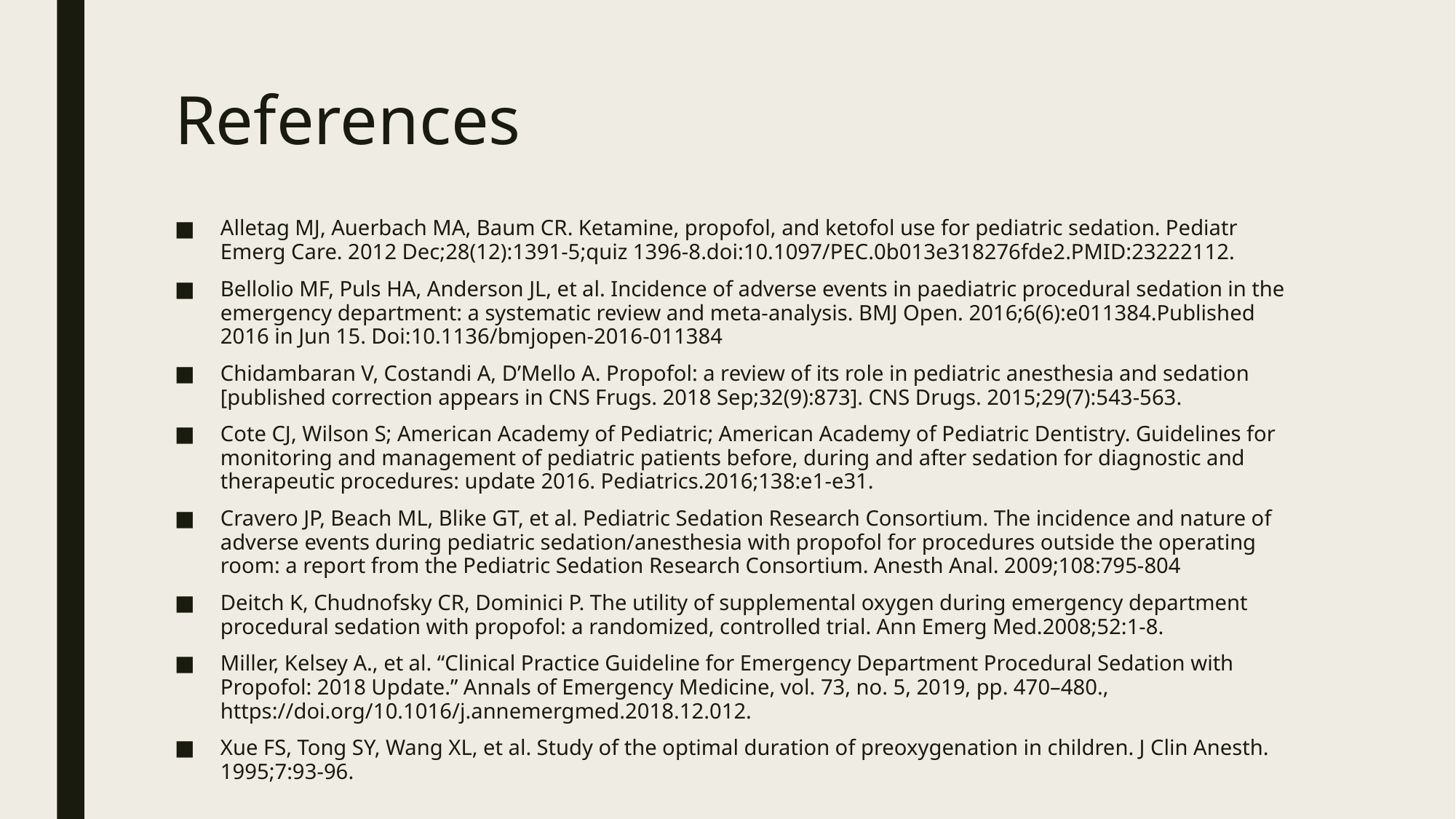

# References
Alletag MJ, Auerbach MA, Baum CR. Ketamine, propofol, and ketofol use for pediatric sedation. Pediatr Emerg Care. 2012 Dec;28(12):1391-5;quiz 1396-8.doi:10.1097/PEC.0b013e318276fde2.PMID:23222112.
Bellolio MF, Puls HA, Anderson JL, et al. Incidence of adverse events in paediatric procedural sedation in the emergency department: a systematic review and meta-analysis. BMJ Open. 2016;6(6):e011384.Published 2016 in Jun 15. Doi:10.1136/bmjopen-2016-011384
Chidambaran V, Costandi A, D’Mello A. Propofol: a review of its role in pediatric anesthesia and sedation [published correction appears in CNS Frugs. 2018 Sep;32(9):873]. CNS Drugs. 2015;29(7):543-563.
Cote CJ, Wilson S; American Academy of Pediatric; American Academy of Pediatric Dentistry. Guidelines for monitoring and management of pediatric patients before, during and after sedation for diagnostic and therapeutic procedures: update 2016. Pediatrics.2016;138:e1-e31.
Cravero JP, Beach ML, Blike GT, et al. Pediatric Sedation Research Consortium. The incidence and nature of adverse events during pediatric sedation/anesthesia with propofol for procedures outside the operating room: a report from the Pediatric Sedation Research Consortium. Anesth Anal. 2009;108:795-804
Deitch K, Chudnofsky CR, Dominici P. The utility of supplemental oxygen during emergency department procedural sedation with propofol: a randomized, controlled trial. Ann Emerg Med.2008;52:1-8.
Miller, Kelsey A., et al. “Clinical Practice Guideline for Emergency Department Procedural Sedation with Propofol: 2018 Update.” Annals of Emergency Medicine, vol. 73, no. 5, 2019, pp. 470–480., https://doi.org/10.1016/j.annemergmed.2018.12.012.
Xue FS, Tong SY, Wang XL, et al. Study of the optimal duration of preoxygenation in children. J Clin Anesth. 1995;7:93-96.
