# Supplementary material for: Pediatric Emergency Medicine Didactics and Simulation (PEMDAS): Pediatric Sedation Complications
Source: MedEdPORTAL. 2024 Feb 13;20:11384. doi: 10.15766/mep_2374-8265.11384 (PMC10861802; doi:10.15766/mep_2374-8265.11384)
Supplement: Supplementary file 1 — Sedation Simulation Cases.docxSedation Simulation Patients.docxCritical Actions Checklist.docxSedation Simulation Equipment.docxSedation Simulation X-Ray Images.docxSedation Simulation Debriefing Materials.docxSedation Simulation Evaluation.docxPropofol and Ketamine.pptx [file mep_2374-8265.11384-s001.zip › A. Sedation Simulation Cases.docx]

| **Appendix A: Sedation Simulation Cases**  Pediatric Emergency Medicine Didactics and Simulation (PEMDAS): Sedation Complications  Authors: Amanda Dupont DO, Daisy Ciener MD MS, Cecilia Monteilh MD, Anita Bharath MD, Anita Thomas MD MPH, Katherine Wolpert MD and Jean Pearce MD MS | | |
| --- | --- | --- |
| **PATIENT #1**  NAME: Adam  AGE: 12 years old  WEIGHT: 40 KG  CHIEF COMPLAINT: Shoulder pain | | **PATIENT #2**  NAME: Sam  AGE: 10 years old  WEIGHT: 30 KG  CHIEF COMPLAINT: Arm injury |
| Brief narrative description of case | The team is working on an overnight shift in the emergency department (ED) and have two patients requiring sedation for procedures.  **Patient #1**: Adam is a 12-year-old male presenting to the ED with shoulder pain. He was playing football this morning and felt a pop in his right shoulder during scrimmage but didn’t seek care until this evening. An X-ray confirmed a right shoulder anterior dislocation. Attempts at reduction including scapular manipulation, prone technique with weights hanging, and the external rotation technique were unsuccessful. He is well appearing with age-appropriate vital signs. He is holding his right forearm close to his body and has a deformity noted at his right shoulder. The team plans for reduction by orthopedic surgery under propofol sedation in the ED. Anticipated initial interventions include obtaining a pre-sedation history, performing a focused pre-sedation physical exam, and obtaining informed consent/assent from the patient’s legal guardian/patient. The team should ensure the room and patient are appropriately prepared with IV access, airway equipment, capnography, pulse oximetry, and cardiac monitoring. The team lead should perform a ‘time out’ prior to the start of the sedation. The patient will require two doses of propofol for adequate sedation. After the second dose of propofol he will develop apnea requiring bag-valve-mask and progressively worsening hypotension requiring a normal saline (NS) bolus. The procedure is completed successfully, and the sedation wears off. Adam wakes up appropriately with normal vital signs and is discharged home.  **Patient #2:** The team then moves to the next patient sedation. The charge nurse brings to the team leader’s attention that there are three patients in the waiting room with displaced forearm fractures who will either need sedation and reduction in the ED or will need to go to the operating room (OR) for general anesthesia and reduction. They presents the team lead with a list of the patients including a 5-year-old female with a forearm deformity and active wheezing in mild respiratory distress (Patient A), a 10-year-old male with forearm deformity with a remote history of bronchiolitis (Patient B), and a 6-year-old with forearm deformity with a history of being born at 23 weeks GA with chronic lung disease on O2 at night and muscular dystrophy with restrictive lung disease (Patient C). The team should recognize that Patient B is appropriate for sedation under ketamine in the ED due to an ASA classification of 1 but that patients A and C are not due to ASA classifications of 3. Anticipated initial interventions include obtaining a pre-sedation history, performing a focused pre-sedation physical exam, and obtaining informed consent/assent from the patient’s legal guardian/patient. The team should ensure the room and patient are appropriately prepared with IV access, airway equipment, capnography, pulse oximetry, and cardiac monitoring. The team lead should perform a ‘time out’ prior to the start of the sedation. Two minutes after ketamine is given, the patient develops laryngospasm and increased salivation with hypoxia to 85% and ETCO2 to 0. Anticipated interventions include jaw thrust with laryngospasm notch pressure and hyperextension of the neck and bag-valve-mask ventilation with appropriate seal. The ETCO2 will continue to read 0 with worsening oxygen saturations to 45%, perioral cyanosis and heart rate decreasing. The team should call a code and administer succinylcholine while continuing airway interventions. The patient will awaken 3-4 minutes after succinylcholine is administered with spontaneous ventilations. The mother will return and the team lead will need to explain the events to her. | |
| Primary Learning Objectives | 1. Understand the indications and contraindications of ED procedural sedation with propofol and ketamine 2. Assign ASA classifications in pediatric patients and recognize which ASA classifications are appropriate for a sedation in the ED 3. Complete the informed verbal consent including sedation plan, associated risks, benefits, and alternatives and obtain a pre-sedation history and perform focused pre-sedation physical exam 4. Demonstrate preparation for ED sedation including obtaining vascular access and selecting and setting up an appropriate size bag-valve-mask (BVM), capnography, suction, pulse oximetry and cardiac monitoring. 5. State and administer correct dosing of sedation medications 6. Recognize and manage complications of propofol including hypoxia, apnea, and hypotension 7. Recognize and manage complications of ketamine including laryngospasm | |
| Critical Actions | Patient #1 (Adam):   1. Obtain verbal consent from the guardian/parent of the patient 2. Explain sedation plan 3. Discuss risks, benefits and alternatives of sedation 4. Determine if patient is appropriate for ED sedation    1. Obtain a pre-sedation history    2. Perform a pre-sedation physical exam    3. Assign an ASA classification 5. Prepare the room and patient appropriately for sedation 6. Obtain vascular access 7. Set up emergency airway equipment including appropriately sized BVM 8. Set up appropriate patient monitoring and supplies (capnography, suction, pulse oximetry, cardiac monitoring) 9. Perform a pre-sedation “time out” with RN, guardian and procedure performing physician present 10. Administer correct doses of propofol 11. Recognize and appropriately manage apnea with airway repositioning and BVM ventilation 12. Recognize and appropriately manage hypotension with a 20 cc/kg NS bolus   Patient #2 (Sam):   1. Assign ASA classifications and determine, based on ASA classification, which patient is appropriate for ED sedation 2. Obtain verbal consent from guardian/parent of the patient 3. Explain sedation plan 4. Discuss risks, benefits and alternative of sedation 5. Confirm the patient is appropriate for ED sedation    1. Obtain a pre-sedation history    2. Perform a pre-sedation physical exam    3. Assign an ASA classification 6. Confirm the room and patient are ready for sedation 7. Double check equipment is available including appropriately sized BVM, pulse oximeter, capnography, cardiorespiratory monitoring, and suction 8. Perform a pre-sedation “time out” with RN, guardian and procedure performing physician present 9. Use closed loop communication at administer correct dose of ketamine 10. Recognize and appropriately manage laryngospasm with: 11. Vigorous airway repositioning with BVM 12. Laryngospasm notch pressure with jaw thrust (Larsen’s maneuver) 13. Administration of succinylcholine 1 mg/kg IV 14. Continued BVM ventilation until the patient has spontaneous respiratory effort and is maintaining airway and saturations | |
| Learner Preparation | Optional Pre/post readings to supplement simulation: Educational power points | |

| Initial Presentation: Patient #1 (Adam) | | | |
| --- | --- | --- | --- |
| Initial vital signs | Weight 40 kg  Heart rate (HR) 90  Oxygen saturation (SpO2) 98%  Blood pressure (BP) 110/72  Respiratory rate (RR) 18  Temperature (T) 37.5 degrees Celsius | | |
| Overall Appearance | A 12-year-old-male is brought in by his parent. He is sitting on a bed, in a hospital gown holding his forearm to his chest and appears uncomfortable. | | |
| Actors and roles in the room at case start | Doctor #1: Team Leader  Nurse #1: Bedside/Sedation Nurse (RN)  Nurse #2: Charge Nurse (RN)  All nurses and doctors can be at any level of training, depending on the audience the simulation is geared towards.  Instructor #1: Simulation instructor who will also act as debriefer  Instructor #2: If a 2^nd^ instructor is available, cast them as “parent,” available to answer questions and assist with debrief. If a 2^nd^ instructor is not available, the facilitator can play the role of parent as well. Can also play the role of orthopedic surgeon. | | |
| HPI | Adam is a 12-year-old male with an anterior shoulder dislocation which the ED team has been unable to reduce who now requires propofol sedation in the ED for reduction with orthopedic surgery.  He was playing football this morning and felt a pop in his right shoulder during the scrimmage. He sat out for the rest of the morning practice. Throughout the day his mother noticed he was unable to do much with his right arm. This evening she asked him to dry the dishes and put them away but he was unable to reach overhead and place them in the cabinet due to restricted range of motion.  After examining him and obtaining x-rays you discovered he had a right anterior shoulder dislocation. You offer an NSAID, Tylenol or opioid for pain relief while you decide on which shoulder reduction technique to attempt first. You attempted scapular manipulation, prone technique with weights hanging, and the external rotation technique. You were unsuccessful and determine that shoulder reduction should be performed under propofol sedation in the ED by orthopedic subspecialty.  SAMPLE history (*if asked*):  Signs/symptoms- Right shoulder pain and limited range of motion  Allergies- none  Meds- none  PMH: none  Last intake: Dinner 8 hours ago  Events preceding- As above.  ROS: negative  Social History: Lives with parents at home. Is in the 7^th^ grade and plays on the football team | | |
| Past Medical/Surgical History  None  Has never been sedated or received general anesthesia.  Does not snore.  No recent or current upper or lower respiratory infections. | Medications  None | Allergies  None | Family History  None  No family history of sedation or anesthesia difficulty |
|  |  |  |  |
| Physical Examination (Primary survey) (Secondary survey)  Weight 40 kg  HR 125  SpO2 99%  BP 135/85  RR 25  T 37.5 degrees Celsius | | | |
| General | Awake, alert, appears mildly uncomfortable, sitting up in bed holding his forearm | | |
| HEENT | Patent airway. No signs of head trauma, pupils are 3mm to 2mm bilaterally, No hemotympanum, nares clear without blood, oropharynx clear | | |
| Neck | Supple, full range of motion, no midline cervical spine tenderness to palpation | | |
| Lungs | Clear to auscultation bilaterally, no stridor or wheezing, no increased work of breathing | | |
| Cardiovascular | Regular rate and rhythm, 2+ radial and pedal pulses, capillary refill 2 seconds | | |
| Abdomen | Abdomen soft, nontender, nondistended, normal bowel sounds, no masses | | |
| Neurological | Glasgow coma scale 15. Pupils 3mm and reactive. Sensation in right upper extremity is intact in all distributions | | |
| Skin | No rash or bruises. Warm, and dry | | |
| Musculoskeletal | Right shoulder with asymmetry and deformity, limited range of motion at shoulder and weak grip strength on the right | | |

| Initial Presentation: Patient #2 (Sam) | | | |
| --- | --- | --- | --- |
| Initial vital signs | Weight 30 kg  HR 110  SpO2 98%  BP 110/72  RR 28  T 37.5 degrees Celsius | | |
| Overall Appearance | A 10-year-old-male brought in by his parent. He is sitting in bed, in a hospital gown holding his forearm against his chest and appears uncomfortable. | | |
| Actors and roles in the room at case start | Doctor #1: Team Leader  Nurse #1: Bedside/Sedation Nurse (RN)  Nurse #2: Charge Nurse (RN)  All nurses and doctors can be at any level of training, depending on the audience the simulation is geared towards.  Instructor #1: Simulation instructor who will also act as debriefer.  Instructor #2: If a 2^nd^ instructor is available, cast them as “parent,” available to answer questions and assist with debrief. If a 2^nd^ instructor is not available, the facilitator can play the role of parent as well. Can also play the role of orthopedic surgeon. | | |
| HPI | Sam is a 10-year-old male with a midshaft angulated and displaced right radius and ulna fracture from a fall off the monkey bars requiring ED sedation for reduction with orthopedic surgery.  He was playing at school and fell onto an outstretched arm. Immediately felt a snap and noticed a deformity. His parent was called who brought him straight here.  He received IN fentanyl in triage and an XR was done showing the fracture.  SAMPLE history (*if asked*):  Signs/symptoms- right forearm pain and deformity, neurovascularly intact  Allergies- none  Meds- none  PMH: none  Last intake: Dinner 8 hours ago  Events preceding- As above.  ROS: negative  Social History: Lives with parents at home. Patient is in the 5^th^ grade. | | |
| Past Medical/Surgical History  None  Has never been sedated or received general anesthesia.  Does not snore.  No recent or current upper or lower respiratory tract infections. | Medications  None | Allergies  None | Family History  None  No family history of sedation or anesthesia difficulty. |
|  |  |  |  |
| Physical Examination (Primary survey) (Secondary survey)  Weight 30 kg  HR 110  SpO2 99%  BP 110/72  RR 28  T 37.5 degrees Celsius | | | |
| General | Awake, alert, appears mildly uncomfortable, sitting up in bed holding his forearm | | |
| HEENT | Patent airway, no signs of head trauma, pupils are reactive 3mm to 2mm bilaterally, no hemotympanum, nares clear without blood, oropharynx clear | | |
| Neck | Supple, full range of motion, no midline cervical spine tenderness to palpation | | |
| Lungs | Clear to auscultation bilaterally, no stridor or wheezing, no increased work of breathing | | |
| Cardiovascular | Regular rate and rhythm, 2+ radial and pedal pulses, capillary refill 2 seconds | | |
| Abdomen | Abdomen soft, nontender, nondistended, normal bowel sounds, no masses | | |
| Neurological | Glasgow coma scale 15. Pupils 3mm and reactive. Sensation in RUE is intact in all distributions | | |
| Skin | No rash or bruises. Warm, and dry | | |
| Musculoskeletal | Right forearm with deformity, bruising and swelling, no skin breaks or skin tenting | | |

| **Instructor Notes - Changes and Case Branch Points** | | |
| --- | --- | --- |
| Intervention / Time Point in Scenario | Change in Case | Additional Information |
| Patient #1 (Adam) | | |
| Parent asks what the plan is to reduce the shoulder/ 0 minutes | Learner should discuss the plan for propofol sedation with the parent including the potential side effects, benefits and alternatives and obtain verbal consent.  Sample script: *“We would like to give your child medication to sedate him so that his dislocated shoulder can be fixed. The medication we use is called propofol. This is a short-acting anesthetic medication that goes in an IV and causes sedation so that your child can be comfortable and we can safely complete the procedure. With any sedation medication there can be side effects which we will prepare for in advance. With propofol, common side effects can include pain at the injection site and nausea and vomiting. More serious but rare side effects include low blood pressure and decreased or even absent breathing. If any of these side effects happen, we are prepared to manage them with fluids, medications and assistance with breathing including intubation. The alternative to not using this medication for sedation would be giving your child some pain medicine and attempting to fix his dislocated shoulder but that may not be effective. What questions do you have for me about the sedation?”* | If not all aspects of consent are discussed by the provider, the parent can ask specific questions about these (i.e. *“what side effects are there?”* or *“what option is there if we don’t want to use this medicine?”*) |
| Pre-sedation history obtained and exam done/ 2 minutes | Learners should:   1. Obtain a pre-sedation history including a history of prior sedation problems, medical history, current medications, allergies, if snores, NPO time and family history of adverse reactions to anesthesia, including malignant hyperthermia. 2. Perform a pre-sedation physical exam including GCS/ awareness, pupillary exam, airway patency, jaw size, mouth opening, Mallampati score, cardiac, pulmonary, and hydration status. 3. Assign an ASA classification | Past medical history: none, no prior sedation or general anesthesia, does not snore  Medications: none  Family history: no issues with sedation or anesthesia  NPO time: 8 hours  HEENT: pupils 3 mm and reactive bilaterally, airway patent, Mallampati score 1 with complete visualization of the palate, unrestricted mouth opening  Pulmonary: lungs clear bilaterally, no increased work of breathing  Cardiac: Regular rate and rhythm, normal S1S2, no murmurs, peripheral pulses +2 and cap refill 2-3 seconds  Neuro: GCS 15, alert and communicative  Can have the RN ask *“What is this patient’s ASA classification?”* if not provided by the learner |
| Preparation for sedation/ 4 minutes | Learner should:   1. Request vascular access 2. Select and set up an appropriate size BVM 3. Request capnography, pulse oximetry, cardiac monitoring and suction be set up and double check this equipment 4. Verify correct loading dose and subsequent doses of propofol with sedation nurse | IV access is obtained on the first attempt.  Capnography, pulse oximetry, cardiac monitoring and suction are set up. |
| Sedation started/ 5 minutes | Learner should:   1. Initiate a ‘time out’ per their institutional policy 2. Consider administration of 20 mg of IV lidocaine 1% without epinephrine immediately before propofol to reduce injection-associated pain 3. Administer propofol 40-60 mg (1-1.5 mg/kg) over 20-30 seconds (or a dose consistent with their institution’s sedation policy) | Pre-sedation vitals:  Weight 40 kg, HR 90, BP 110/72, RR 18, SpO2 98%, ETCO2 35  If the learner doesn’t give lidocaine, can have the RN ask *“I know propofol usually burns, is there something we can give to help this?”*  Patient is sedated with a GCS of 6 (E1V1M4) within 60 seconds and the parent leaves the room. |
| Repeat dose/7 minutes | The orthopedic provider is having difficulty with the reduction and the patient is waking up.  Learner should:   1. Request a second dose of 20mg (0.5 mg/kg) of IV propofol (or a dose consistent with their institution’s sedation policy) 2. Explain why not to give an IV opiate with propofol (airway loss, hypotension, etc.) | HR 76, Sat 96%, BP 100/64, RR 16, ETCO2 35  Prior to the second dose of medication, the patient is quietly moaning and resisting the orthopedic provider’s efforts at reduction. Can have the RN ask *“This patient seems to be in a lot of pain, shouldn’t we give some morphine or something?”* |
| Apnea/ 8 minutes | Learner should:   1. Administer BVM ventilation 2. Apply a jaw thrust | HR 70, Sat 85%, BP 100/64, RR 0, ETCO2 0  Patient’s RR declines over 30 seconds to 0.  With BVM ventilation, the SpO2 increases to 99% and ETCO2 to 35.  After 2 minutes of BVM ventilation, the patient begins spontaneously breathing and the RR improves to 16. |
| Hypotension / 10 minutes | Learner should administer a 20 cc/kg normal saline bolus rapidly. | BP is 80/60, HR 76, RR 16, ETCO2 35, SpO2 99%  Can have the RN ask *“how quickly do you want me to give the fluids?”* if this is not specified.  BP increases to 110/72 after NS bolus initiated.  Orthopedic provider has successfully reduced the dislocation after the bolus is initiated. |
| End patient #1 case/11 minutes | Learner should explain events to mother and patient including:   1. Apnea requiring BVM 2. Hypotension requiring fluids | HR 110, BP 100/72, RR 16, EtOC2 35, SpO2 99%  The patient awakens and his mother returns to the room. Neither have any additional questions after being updated by the learner. |
| Patient #2 (Sam) | | |
| Charge RN asking to discuss sedation plans/13 minutes | Learner should discuss three potential sedation patients and assign ASA classifications to determine which one is appropriate for ED sedation.  Learner should tell charge RN that patient ‘B’ should be roomed for procedural sedation in the ED.. | Charge RN can provide the learner with a list of the three patients (*Appendix B*) with fractures in the waiting room and ask which one he/she should room for an ED sedation.  Learner should assign ASA classifications to all three patients and recognize that only patient ‘B’ is an ASA classification of 1 and patients ‘A’ and ‘C’ are ASA classifications of 3. |
| Parent asks what the plan is to fix the forearm fracture/ 15 minutes | Learner should discuss the plan for ketamine sedation with the mother including the mechanism of action of ketamine, potential side effects, benefits and alternatives and obtain verbal consent.  Sample script: *“We would like to give your child medication to sedate him so that his fractured arm can be fixed. The medication we use is called ketamine. This is an anesthetic medication that goes in an IV and provides sedation so that your child can be comfortable and we can safely complete the procedure. With any sedation medication there can be side effects which we will prepare for in advance. With ketamine, common side effects can include nausea and vomiting and twitching of the muscles and eyes which is temporary. More serious but rare side effects can include trouble breathing, decreased or even absent breathing, and some distress and anxiety when waking up. If any of these side effects happen, we are prepared to manage them with medications and assistance with breathing including intubation. The alternative to not using this medication for sedation would be going to the operating room for repair of this injury under general anesthesia. What questions do you have for me about the sedation?”* | If not all aspects of consent are discussed by the provider, the parent can ask specific questions about these (i.e. *“what side effects are there?”* or *“what option is there if we don’t want to use this medicine?”*) |
| Pre-sedation history obtained and exam done/ 17 minutes | Learners should:   1. Obtain a pre-sedation history including a history of prior sedation problems, medical history, current medications, allergies, if snores, NPO time and family history of difficult sedations. 2. Perform a pre-sedation physical exam including GCS/ awareness, pupillary exam, airway patency, jaw size, mouth opening, Mallampati score, cardiac, pulmonary, and hydration status. 3. Assign an ASA classification | Past medical history: none, no prior history of sedation, does not snore  Medications: none  Family history: no issues with sedation or anesthesia  NPO time: 8 hours  HEENT: pupils 3 mm and reactive bilaterally, airway patent, Mallampati score 1 with complete visualization of the palate, unrestricted mouth opening  Pulmonary: lungs clear bilaterally, no increased work of breathing  Cardiac: Regular rate and rhythm, normal S1S2, no murmurs, peripheral pulses +2 and cap refill 2-3 seconds  Neuro: GCS 15, alert and communicative  Can have RN ask *“What is this patient’s ASA classification?*” if not provided by the learner |
| Sedation started/ 19 minutes | Learner should:   1. Double-check set up of appropriate size BVM, capnography, pulse oximetry, cardiac monitoring and suction 2. Verify correct loading dose and subsequent doses of ketamine with sedation nurse 3. Initiate a ‘time out’ per their institutional policy 4. Administer ketamine 30-45 mg (1-1.5mg/kg) IV over 60-90 seconds (or a dose consistent with their institution’s sedation policy) | Sedation RN should tell the learner that the IV is already placed and the room is already set up for sedation.  Pre-sedation vitals:  HR 120, BP 110/72, RR 20, SpO2 98%, ETCO2 35  Parent leaves the room after the loading dose of ketamine is given.  1 minute after Ketamine vitals:  HR 125, BP 130/90, RR 20, SpO2 98%, ETCO2 35, GCS 6 (E1V1M4) |
| Laryngospasm/21 minutes | Learner should:   1. Suction 2. Perform vigorous jaw thrust with laryngospasm notch pressure(Larsen’s maneuver) and neck hyperextension 3. Apply BVM with continuous positive pressure | HR 125, BP 130/90, RR 0, SpO2 85%, ETCO2 0  Patient develops laryngospasm; increased salivation, oxygen saturation slowly downtrends from 98% to 85% and continues to fall. End tidal will slowly decline to 0 and then flat line. |
| Continued laryngospasm/ 22 minutes | Learner should:   1. Activate a code 2. Administer 30 mg (1 mg/kg) IV succinylcholine 3. Maintain jaw thrust, laryngospasm notch pressure and BVM 4. May consider atropine for bradycardia which can worsen with succinylcholine | HR 80, BP 130/90, RR 0, SpO2 75%, ETCO2 0  Patient now with perioral cyanosis. HR is slowly down trending but not below 60. Note: succinylcholine can worsen bradycardia, consider atropine.  SpO2 increases to 99% and ETCO2 to 35 thirty seconds after succinylcholine. |
| End of case/ 32 minutes | Learner should explain events to mother and patient including:   1. Laryngospasm requiring interventions 2. Explain to the parent that the procedure could not be completed due to adverse airway event and the learner recommends anesthesia sedation in the OR. | HR 120, BP 130/90, RR 20, SpO2 99%, ETCO2 35  Patient breathing spontaneously, eyes open and awake but drowsy. |

**Ideal Scenario Flow**

The team should start the first case by obtaining informed consent from the parent, obtaining a pre-sedation history and performing a focused pre-sedation physical exam. The team should then ensure the room and patient are appropriately prepared with IV access, airway equipment, capnography, pulse oximetry, and cardiac monitoring. The team lead should perform a ‘time out’ prior to the start of the sedation. The team can consider a dose of IV lidocaine for injection site pain prior to the first bolus of propofol. The team will need to administer a second dose of propofol in order to achieve adequate sedation. Following the second dose the patient develops apnea requiring airway repositioning and BVM ventilation after which he begins breathing spontaneously. The patient will then develop hypotension requiring a NS bolus. The procedure is completed successfully and the team should explain the complications to the patient and his parent. The team then moves to the next patient sedation. The team lead will select patient ‘B’ from a list of three patients with displaced fractures as the patient with an ASA classification of 1 who is appropriate for ED sedation. The team should start the second case by obtaining informed consent from the parent, obtaining a pre-sedation history and performing a focused pre-sedation physical exam. The team lead should perform a ‘time out’ prior to the start of sedation. After the patient develops laryngospasm with hypoxia the team should perform a jaw thrust with laryngospasm notch pressure, hyperextension of the neck and BVM with appropriate seal. The vital signs will continue to decline and the team should call a code and administer succinylcholine while continuing airway interventions. The patient will awaken 3-4 minutes after succinylcholine is administered with spontaneous ventilations. The parent will return and the team will need to explain the events to her ending the case.

**Anticipated Management Mistakes**

1. **Incorrect patient selected for sedation**: Provider selects a patient with an ASA classification of 3 for sedation in the ED. The charge RN can prompt the provider to select the patient with an ASA classification of I for ED sedation by asking questions like *“the triage RN heard wheezing on that patient, are you sure we are ok to sedate in the ED?”* or *“this patient has a pretty complicated pulmonary history, are you sure we can sedate in the ED?”*
2. **Incorrect dosing of ketamine or propofol:** Incorrect dosing creating either too deep of a sedation with subsequent complications or providing too mild of sedation requiring frequent re-dosing. If the patient is under dosed, then the patient starts moving and cries with manipulation of the forearm or shoulder by the procedural provider. If the provider administers too large of a dose of sedative then the patient pushes the boundary of moderate to deep sedation and takes longer to awaken with bag mask ventilation/ air way repositioning. Blood pressure returns to normal after two normal saline boluses are administered.
3. **Failure to recognize complications/ side effects of sedation medications**: For example, the patient becomes profoundly hypotensive during sedation with propofol. RN to guide provider and hint at vital signs/ become worried in the room
4. **Failure to administer a paralytic as a reversal of ketamine**: If the provider continues to BVM ventilate the patient to assist in ventilation without administering succinylcholine. RN can prompt the provider by saying *“is there anything else we can do?”* or *“is there any medication we can give to help?”*
5. **Provider intubates sedation patients**: If team elects to intubate sedation patients, can have them do this and then discuss in the debrief that if this occurs they may just be able to provide BVM ventilation while waiting for the sedation to wear off
