# Supplementary material for: Pediatric Emergency Medicine Didactics and Simulation (PEMDAS): Pediatric Sedation Complications
Source: MedEdPORTAL. 2024 Feb 13;20:11384. doi: 10.15766/mep_2374-8265.11384 (PMC10861802; doi:10.15766/mep_2374-8265.11384)
Supplement: Supplementary file 1 — Sedation Simulation Cases.docxSedation Simulation Patients.docxCritical Actions Checklist.docxSedation Simulation Equipment.docxSedation Simulation X-Ray Images.docxSedation Simulation Debriefing Materials.docxSedation Simulation Evaluation.docxPropofol and Ketamine.pptx [file mep_2374-8265.11384-s001.zip › B. Sedation Simulation Patients.docx]

**Appendix B: Waiting room patients for patient #2 case**

| **Patient A:** Kim is a 5-year-old female with a history of moderate persistent asthma presenting to triage with an obvious deformity of the right forearm secondary to jumping off a platform on the playground while playing tag.  Vital signs and exam:  Weight 18 kg \| HR 115 \| BP 110/70 \| RR 28 \| SPO2 97%  She is in mild respiratory distress with diffuse end-expiratory wheezing throughout and moderately increased work of breathing and diminished aeration throughout with a PASS* of 3. She has an obvious deformity of the right forearm with 2+ radial pulses. Capillary refill < 2 seconds with warm extremities |
| --- |
| **Patient B:** Sam is a 10-year-old male with a history of bronchiolitis at 6 months of age presenting from school with an obvious deformity to his left forearm secondary to falling off the monkey bars.  Vital signs and exam:  Weight 30 kg \| HR 110 \| BP 110/ 72 \| RR 20 \| SPO2 99%  He is sitting in a triage chair holding his arm and appears uncomfortable due to pain. His heart rate is regular and his lungs are clear to auscultation. He has an obvious deformity of the left forearm with 2+ radial pulses and capillary refill < 2 seconds with warm extremities. |
| **Patient C:** Nick is a 6-year-old male, ex 23-week gestational age, with chronic lung disease from bronchopulmonary dysplasia requiring 1 L of supplemental oxygen at night and muscular dystrophy with restrictive lung patterns presenting with an obvious forearm deformity secondary to falling out of his wheelchair and hitting a side table on the way to the ground.  Vitals and exam:  Weight 20 kg \| HR 90 \| BP 100/ 74 \| RR 20 \| SPO2 96%  He is sitting in his wheelchair in mild distress due to pain with coarse breath sounds bilaterally with an obvious deformity of the left forearm. His radial pulses are 2+ with capillary refill 2 seconds with cool extremities. |

*PASS = Pediatric Asthma Severity Score

| Clinical Finding | Definition | 0 | 1 | 2 |
| --- | --- | --- | --- | --- |
| Wheezing | High-pitched expiratory sound heard by auscultation | None or mild | Moderate | Severe wheezing or absent wheezing due to poor air exchange |
| Air entry | Intensity of inspiratory sounds by auscultation | Normal or mildly diminished | Moderately diminished | Severely diminished |
| Work of breathing | Observed use of accessory muscle, retractions or in-breathing | None or mild | Moderate | Severe |

Adopted from: Gorelick et al., Performance of Novel Clinical Score, the Pediatric Asthma Severity Score (PASS), in the Evaluation of Acute Asthma. *Academic Emergency Medicine*. 11 (1): 2004. 10-18.
