# Supplementary material for: Pediatric Emergency Medicine Didactics and Simulation (PEMDAS): Pediatric Sedation Complications
Source: MedEdPORTAL. 2024 Feb 13;20:11384. doi: 10.15766/mep_2374-8265.11384 (PMC10861802; doi:10.15766/mep_2374-8265.11384)
Supplement: Supplementary file 1 — Sedation Simulation Cases.docxSedation Simulation Patients.docxCritical Actions Checklist.docxSedation Simulation Equipment.docxSedation Simulation X-Ray Images.docxSedation Simulation Debriefing Materials.docxSedation Simulation Evaluation.docxPropofol and Ketamine.pptx [file mep_2374-8265.11384-s001.zip › D. Sedation Simulation Equipment.docx]

**Appendix D: Simulation Equipment Preparation**

Not all medications or equipment are necessary for the simulation but reflect what is commonly used in pediatric emergency departments for the care of patients.

Personal Protective Equipment

- Staff gowns
- Gloves
- Masks and face shields

Simulated Medications

- Epinephrine
- Etomidate
- Fentanyl
- Ketamine
- Lidocaine
- Lorazepam
- Midazolam
- Morphine
- Norepinephrine
- Normal Saline/Lactated Ringers
- Propofol
- Rocuronium
- Succinylcholine

Equipment

- Simulator manikin in hospital gown or clothing, on bed
- Monitor with NIBP, HR, RR, Oxygen saturation, temperature and ETCO2 monitor
- Blood Pressure cuff, heart rate monitor leads, oxygen saturation probe, defibrillator cables and ETCO2 cannula
- Oxygen source (on wall or cylinder)
- Bag-valve-mask system with multiple size masks
- Nasal cannula
- Oxygen masks - simple and non-rebreather
- Suction device
- Nasal, and oral airways, multiple sizes
- LMAs, multiple sizes
- Shoulder roll
- Endotracheal tubes- 4.0, 4.5, 5.0, 5.5, 6.0, 6.5 cuffed or uncuffed with stylets
- Laryngoscope, Miller and Mac blades, multiple sizes
- Nasogastric tube(s)
- Stethoscopes
- IV/Angiocath, various sizes
- IV tubing
- IV pumps, pressure bags
- Syringes, multiple sizes
- Code cart
- CPR stool, backboard
- Defibrillator / AED
