# Supplementary material for: Pediatric Emergency Medicine Didactics and Simulation (PEMDAS): Pediatric Sedation Complications
Source: MedEdPORTAL. 2024 Feb 13;20:11384. doi: 10.15766/mep_2374-8265.11384 (PMC10861802; doi:10.15766/mep_2374-8265.11384)
Supplement: Supplementary file 1 — Sedation Simulation Cases.docxSedation Simulation Patients.docxCritical Actions Checklist.docxSedation Simulation Equipment.docxSedation Simulation X-Ray Images.docxSedation Simulation Debriefing Materials.docxSedation Simulation Evaluation.docxPropofol and Ketamine.pptx [file mep_2374-8265.11384-s001.zip › E. Sedation Simulation X-Ray Images.docx]

**Appendix E: X-ray Images**

Patient #1 (Adam): Anterior Shoulder Dislocation


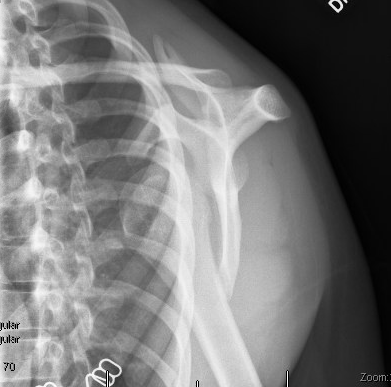


Author owned image


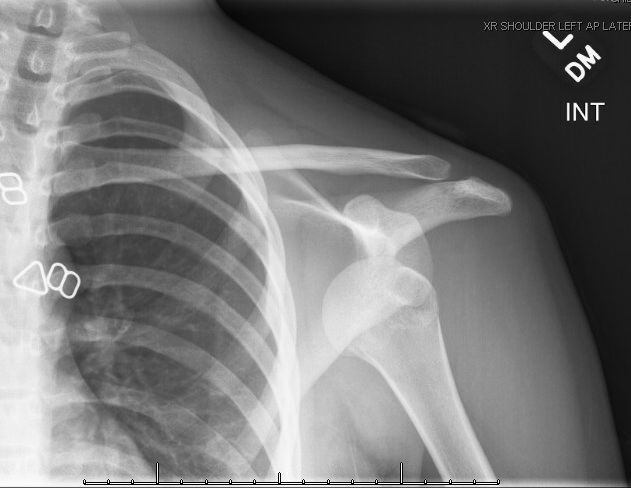


Author owned image


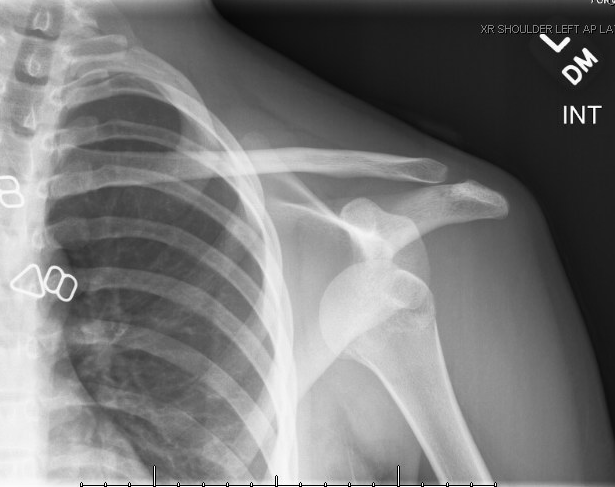


Author owned image

Patient #2 (Sam): Left Forearm Fracture


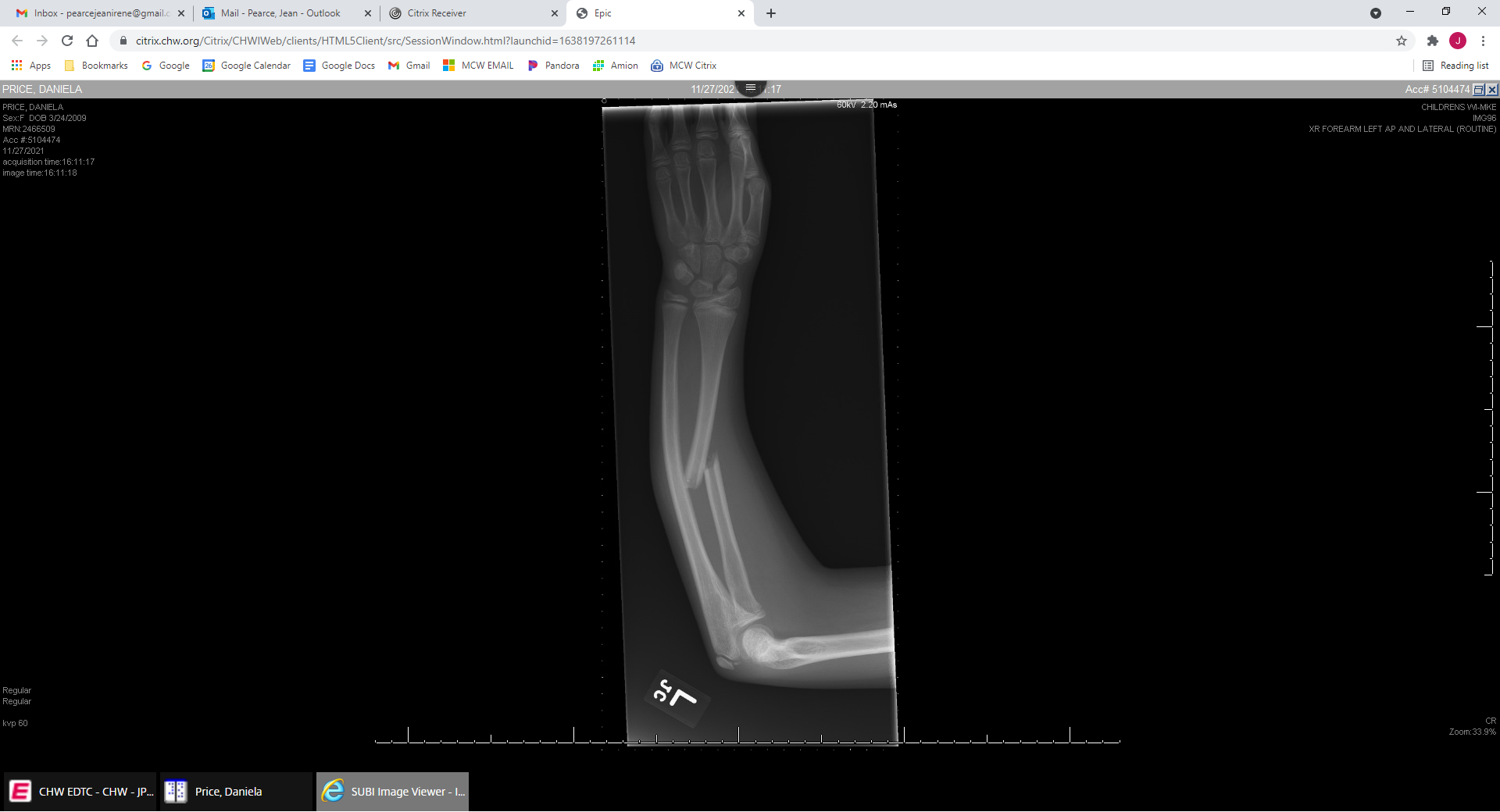

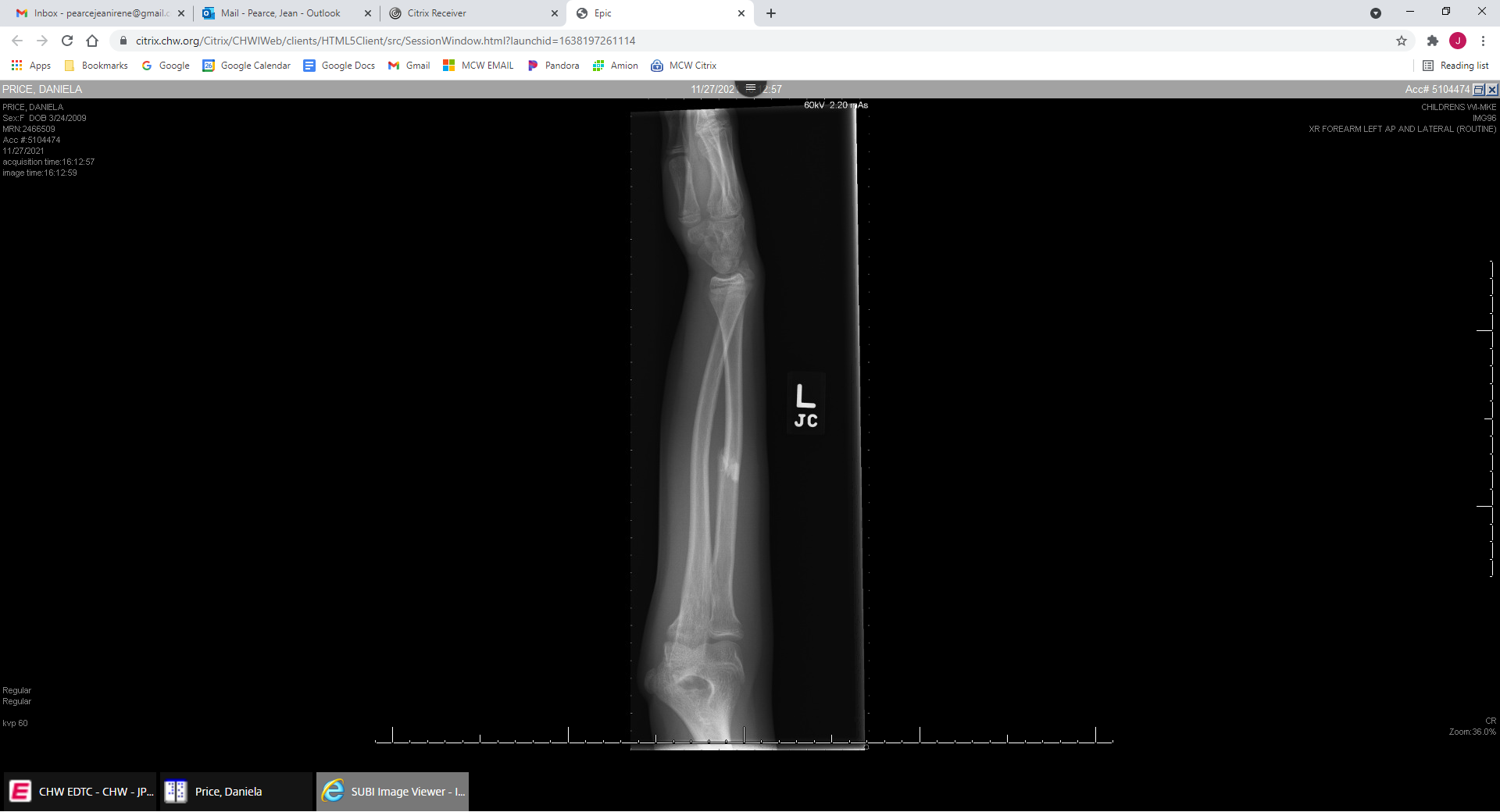
Author owned image Author owned image
