# Supplementary material for: Pediatric Emergency Medicine Didactics and Simulation (PEMDAS): Pediatric Sedation Complications
Source: MedEdPORTAL. 2024 Feb 13;20:11384. doi: 10.15766/mep_2374-8265.11384 (PMC10861802; doi:10.15766/mep_2374-8265.11384)
Supplement: Supplementary file 1 — Sedation Simulation Cases.docxSedation Simulation Patients.docxCritical Actions Checklist.docxSedation Simulation Equipment.docxSedation Simulation X-Ray Images.docxSedation Simulation Debriefing Materials.docxSedation Simulation Evaluation.docxPropofol and Ketamine.pptx [file mep_2374-8265.11384-s001.zip › F. Sedation Simulation Debriefing Materials.docx]

**Appendix F: Sedation Simulation Debriefing Materials**

We believe that the debrief is an important experience for all people involved in the simulation experience – educators, instructors, facilitators, and learners – to re-examine the simulation experience with the goal of fostering the development of clinical judgment and critical thinking skills. It is meant to encourage participants’ reflective thinking and provides an opportunity for the exchange of feedback.^1^

Important principles to consider when preparing for the debrief:

- What teaching point(s) do you want the learners to take away?
- How will you circle back to the learning outcomes/objectives?
- Take a stance of *curiosity*, rather than judgment. Ask questions to get at what the learners were thinking and avoid assuming that a ‘mistake’ is always related to a knowledge deficit.

General Debrief Structure

Our debriefing is modeled after PEARLS^2-3^ and the method created by the Center for Medical Simulation. The debrief is broken into five distinct phases.

1. Preview: Sets the stage and prepares the learners for the debriefing activity.

- *“I’d like to take the next 30 minutes to talk as a group about this scenario. We’ll start with your initial reactions, then discuss the case in more detail, and end with a summary and key take-aways.”*

1. Reactions: Provides the opportunity for learners to voice feelings and “clear the air,” which in turn will allow participants to move on toward further discussion and provide a better environment for learning to occur.

- *“How did you feel when the patient undergoing ketamine sedation had a laryngospasm?”*
- *“What were your initial reactions when you set up the room for the sedation?”*
- *“First thing off the top of your head when you saw the patient undergoing a propofol sedation was hypotensive?”*

1. Description: It is important to make sure everyone is on the same page in order to avoid confusion and facilitate learning.

- *“Can someone please summarize the Ketamine sedation case, including covering ASA classifications, dosing, redosing, complications and management of ketamine complications?”*
- *“To summarize, this case was …”*

1. Analysis/Understanding: The objectives of this phase are to explore participants’ perspectives on the scenario and help them move to a new understanding and develop new skills. There are many different debriefing approaches and styles that can be employed for this stage. Facilitators may use open-ended questions to guide learners toward self-assessment or use more directive feedback to ensure learning objectives are met within time constraints. Most often a combination of strategies is employed based on various factors including time constraints and the experience levels of the learners and facilitators.

- *“Tell me more about appropriate lidocaine dose and why this is important”*
- *“I wonder what you were thinking when the patient went apneic”*
- *“I’m curious about how you selected the appropriate patient for sedation in case 2”*
- *“What things did your team do well? What things could your team do differently next time?”*
- *“I noticed you did [action]. Next time you may want to consider [alternate action] because [rationale].*

1. Summary/Application: The goal of this stage is to identify key take-aways and tie them back to clinical applications. These take-aways are your opportunity to reinforce your learning objectives and also time for the learners to voice key points they identified in the case. These could include medical, technical, teamwork and communication skills. Please refer to the critical actions checklist to help discuss your takeaway points.

- *“During this debrief we’ve talked about ‘X’ and ‘Y’, and I’d like to end with your key takeaways.”*

References

1. *Lopreiato JO (Ed). (2016). Healthcare simulation dictionary. Rockville, MD: Agency for Healthcare Research and Quality; 2016. AHRQ Publication No. 16(17)-0043.*
2. *Eppich W and Cheng A. Promoting Excellence and Reflective Learning in Simulation (PEARLS): Development and Rationale for a Blended Approach to Health Care Simulation Debriefing. Sim in Healthcare. 2015:10 (2): 106-115.*
3. *Center for Medical Simulation. 2019. https://harvardmedsim.org/*

**Facilitator Notes:**
