# Supplementary material for: Pediatric Emergency Medicine Didactics and Simulation (PEMDAS): Pediatric Sedation Complications
Source: MedEdPORTAL. 2024 Feb 13;20:11384. doi: 10.15766/mep_2374-8265.11384 (PMC10861802; doi:10.15766/mep_2374-8265.11384)
Supplement: Supplementary file 1 — Sedation Simulation Cases.docxSedation Simulation Patients.docxCritical Actions Checklist.docxSedation Simulation Equipment.docxSedation Simulation X-Ray Images.docxSedation Simulation Debriefing Materials.docxSedation Simulation Evaluation.docxPropofol and Ketamine.pptx [file mep_2374-8265.11384-s001.zip › G. Sedation Simulation Evaluation.docx]

**Appendix G: Sedation Simulation Evaluation**

Please answer each question based on your simulation experience.

Please select your role:

- Medical student
- Pediatric Resident
- Emergency Medicine Resident
- PEM fellow
- PEM attending
- Other

Please write your institution/instructor: _____________________________________________________

|  | **Strongly disagree** | **Somewhat disagree** | **Neither agree nor disagree** | **Somewhat agree** | **Strongly agree** |
| --- | --- | --- | --- | --- | --- |
| The simulation scenario was realistic |  |  |  |  |  |
| The simulation scenario was clinically relevant. |  |  |  |  |  |
| The simulation helped me improve my overall comfort level in caring for critically ill patients. |  |  |  |  |  |
| The debrief provided valuable learning. |  |  |  |  |  |
| The debrief session was a safe and supportive environment. |  |  |  |  |  |

How could the simulation have been changed to make it better for your learning?

What is one learning point that you will take away from the simulation?

Do you have any additional comments or concerns about the simulation?
