# Supplementary material for: Pediatric Emergency Medicine Didactics and Simulation (PEMDAS): Pediatric Sedation Complications
Source: MedEdPORTAL. 2024 Feb 13;20:11384. doi: 10.15766/mep_2374-8265.11384 (PMC10861802; doi:10.15766/mep_2374-8265.11384)
Supplement: Supplementary file 1 — Sedation Simulation Cases.docxSedation Simulation Patients.docxCritical Actions Checklist.docxSedation Simulation Equipment.docxSedation Simulation X-Ray Images.docxSedation Simulation Debriefing Materials.docxSedation Simulation Evaluation.docxPropofol and Ketamine.pptx [file mep_2374-8265.11384-s001.zip › _Educational Summary Report_11384.pdf]

MedEdPORTAL® The AAMC Journal of Teaching and Learning Resources

Original Publication Open Access

Pediatric Emergency Medicine Didactics and Simulation (PEMDAS): Pediatric Sedation Complications

Amanda Dupont, DO*, Daisy Ciener, MD, MS, Cecilia Monteilh, MD, Anita Bharath, MD, Anita Thomas, MD, MPH, Katherine Wolpert, MD, Jean Pearce, MD, MS

*Corresponding author: adupont@mcw.edu

Abstract

Introduction: Ketamine and propofol are commonly used agents for sedation in the pediatric emergency department (PED). While these medications routinely provide safe sedations, there are side effects providers should be able to recognize and manage. Currently, no pediatric sedation simulations exist in the literature. **Methods:** We created two sedation simulation cases for learners, including pediatric emergency medicine (PEM) fellows, working in the PED: case 1, a 12-year-old male with a shoulder dislocation requiring reduction under propofol sedation, and case 2, a forearm fracture requiring reduction under ketamine sedation. Learner actions included setting up equipment for sedations, dosing medications correctly, and managing complications. Additionally, in case 2, learners assigned an American Society of Anesthesiologists classification and selected the appropriate candidate for PED sedation from amongst three patients. A debrief followed the cases. Next, a didactic presentation reinforced concepts discussed in the debrief. Participants then completed an evaluation of the simulation. **Results:** Fifty-eight emergency medicine residents and PEM fellows across four sites at three institutions participated. Participants scored the simulations and the debriefing session on a 5-point Likert scale. Learners rated the scenario as clinically relevant ($M = 4.37$) and effective at improving their comfort level in caring for critically ill patients ($M = 4.36$). Learners felt the debrief provided valuable learning ($M = 4.40$) and was a safe learning environment ($M = 4.50$). **Discussion:** These cases can be utilized as resources for learners in any emergency department and can be tailored to any training background of learner providing sedation.

Keywords
Apnea, Bag Valve Mask Ventilation, Consent and Assent, Hypotension, Ketamine, Laryngospasm, Propofol, Sedation, Emergency Medicine, Pediatric Emergency Medicine, Simulation

Educational Objectives

By the end of this session, participants will be able to:

1. List both indications and contraindications of emergency department (ED) procedural sedation with propofol and ketamine.
2. Know American Society of Anesthesiologists classifications, including patients who are appropriate for sedation in the ED.
3. Complete the informed verbal consent and obtain a presedation history and physical exam.
4. Demonstrate preparation for ED sedation, including obtaining vascular access and selecting and setting up an appropriately sized bag valve mask, capnography, pulse oximetry, and cardiac monitoring.
5. Know the correct dosing/redosing of sedation medications.
6. Manage complications of propofol.
7. Manage complications of ketamine.

Citation:
Dupont A, Ciener D, Monteilh C, et al. Pediatric Emergency Medicine Didactics and Simulation (PEMDAS): pediatric sedation complications. *MedEdPORTAL*. 2024;20:11384.
https://doi.org/10.15766/mep_2374-8265.11384

Introduction

Procedural sedations in the pediatric emergency department (PED) are commonly performed using either propofol or ketamine. Sedation medications are provider dependent based on desired analgesic effect, availability of the medication in a provider's institution, and the provider's comfort level with the medication. Identifying who is safe for sedation in the emergency department (ED), appropriately setting up the room for sedation, obtaining informed verbal consent, and being familiar with the side effects

Copyright © 2024 Dupont et al. This is an open-access publication distributed under the terms of the Creative Commons Attribution license.

of ketamine and propofol and how to manage these side effects are all important aspects of providing the best patient care in the ED.

Ketamine is a dissociative anesthetic that also provides analgesia and is commonly used for procedures such as fracture reductions that require both properties.[1,2] Propofol is a short-acting, soy-based, IV, nonanalgesic sedative. It acts on neuronal lipid membranes as a GABA agonist, producing rapid and brief sedation in under 10 minutes.[1,3] Each institution may differ in the dosing of these sedatives. For children 3 years and younger, 2.0 mg/kg/dose of propofol can be used as an initial dose. For children 4 years and older, one can use 1.5 mg/kg/dose.[1,3,4] Titration bolus doses range from 0.5 mg/kg to 1.0 mg/kg every 1-3 minutes as needed.[1,4] With each subsequent propofol bolus dose, the body tissues become saturated, thus delaying both the distribution of serum propofol and its clearance rate. Propofol is often administered after 0.5 mg/kg, maximum of 50.0 mg, of IV lidocaine has been administered due to the initial pain with propofol injection.[4] Similar to propofol, there is a range of ketamine dosing that is provider dependent. IV ketamine is dosed at 1.0-1.5 mg/kg, with the initial maximum dose of 100.0 mg. Repeat doses of 0.5 mg/kg to 1.0 mg/kg 10 minutes after the initial dose can be administered.[1,2]

Common side effects of ketamine include hypersalivation, hypertension, nystagmus, emesis, and emergence delirium.[1,2] Propofol-associated hypotension is also a well-documented side effect[2,3,5] and is more commonly observed in patients with depleted intravascular volume. Hypotension is usually transient and self-resolving or responds to IV fluids.[5] Although rare, ketamine-induced laryngospasms and propofol-induced hypoxia and apnea do occur. All are life-threatening and need to be acted on promptly. To manage laryngospasms, positive pressure ventilation with a jaw thrust and laryngospasm notch pressure can be performed. Ultimately a paralytic, preferably succinylcholine 1.0 mg/kg IV or 4.0 mg/kg IM, can be administered if the above interventions do not work.[2] Corrective steps for propofol-induced apnea include stimulation, airway repositioning, supplemental oxygen, and bag valve mask ventilation.[2] Supplemental preoxygenation is recommended for propofol sedations to improve periods of normal oxygenation during respiratory depression or apnea.[6,7] Interventions for these low-frequency but higher-acuity situations can be adequately practiced via simulation.

Methods

These simulation cases (Appendix A) were designed by pediatric emergency medicine (PEM) providers to augment the learner's foundation of sedation knowledge. Through participation in each simulation, learners demonstrated how to take a presedation history, perform a presedation exam, assess American Society of Anesthesiologists (ASA) classification,[8] prepare the room for sedation, perform a time-out, and appropriately dose and redose two common sedation medications used in the ED. Learners were able to recognize common and life-threatening side effects of propofol and ketamine and demonstrated appropriate management of each. The simulations and debrief required around 2 hours to complete; each case ran around 30 minutes, and the debrief sessions required around 30 minutes. Appendices B-H were used in conjunction with Appendix A.

Appendix B detailed three different patients—patients A, B, and C—each with differing histories, physical exams, and ASA classifications. This appendix was created for simulation case 2 to help learners triage appropriate patients for moderate sedation in the ED. A critical actions checklist modeled off emergency medicine (EM) procedural sedation standards of care at our three institutions was written for each simulation (Appendix C) to ensure the educational objectives were met. Supplemental images of a shoulder dislocation and a forearm fracture for cases 1 and 2 were provided in Appendix E. Debriefing was conducted immediately following both simulations in a safe environment using the Promoting Excellence and Reflective Learning in Simulation (PEARLS) debriefing framework.[9] Appendix F contained sedation simulation debriefing materials. We asked all participants across the three institutions to complete an evaluation (Appendix G) for feedback. A didactic, PowerPoint-based presentation on propofol and ketamine (Appendix H) was created to distribute before or after simulations.

We implemented this simulation during PEM teaching sessions for a variety of PEM providers including scheduled weekly and monthly education days and orientation for newly starting trainees. The groups were made up of various levels of training, including fellows (PGY 4-PGY 6), residents (PGY 1-PGY 3), and advanced practice providers (APPs).

Equipment/Environment

Each simulation used a high-fidelity adolescent mannequin and was conducted either in a simulation lab meant to embody a PEM department room or in situ in the actual ED. Appropriate equipment, simulated medications, and personal protective equipment were provided (checklist in Appendix D). Each scenario started with a patient without IV access who required sedation for a procedure. The ED room was not set up for a sedation, and so, the provider had to adequately prepare it. Both

Copyright © 2024 Dupont et al. This is an open-access publication distributed under the terms of the Creative Commons Attribution license.

cases were discussed and reviewed amongst the PEM physicians who ran the simulation prior to the simulations in order to help standardize the scenarios across the three institutions.

Personnel
These simulations were designed to accommodate up to 10 learners and targeted medical personnel working in a PED, including PEM fellows, APPs, PEM faculty, and pediatric and EM residents. When the simulations had enough learners to cover key roles, extra personnel functioned as bedside nurse, patient's parent, and proceduralist. One to two simulation instructors ran the simulation and the debrief afterward.

Implementation
Most learners had some familiarity with procedural sedation in the PED, although there was no expected specific knowledge requirement. The first scenario (Appendix A) began with a 12-year-old male requiring a propofol sedation for an anterior shoulder dislocation. The team planned for reduction by orthopedic surgery under propofol sedation in the ED. Presedation history, focused presedation physical exam, and informed consent/assent were obtained by a sedationist/lead. The team ensured that the room was set up and the patient appropriately prepared for sedation. The team lead performed a time-out prior to the start of the sedation. The patient required two doses of propofol for adequate sedation. After the second dose of propofol, the patient developed apnea, which required a bag valve mask, and progressively worsening hypotension, which required a normal saline bolus or lactated ringers. The procedure was completed successfully, and the patient was discharged home.

The team then moved to the next patient sedation (case 2 in Appendix A). The team was required to select from a list of patients (Appendix B) which one was most appropriate for ED procedural sedation based on history and exam findings, coming up with an ASA level. The team selected which patient was appropriate for sedation under ketamine in the ED. Similar to the prior simulation, initial interventions included obtaining a presedation history, performing a focused presedation physical exam, obtaining informed consent/assent from the patient's legal guardian/patient, ensuring the room and patient were appropriately prepared for sedation, and performing a time-out prior to the start of the sedation. Shortly after ketamine was given, the patient developed laryngospasms and increased salivation with hypoxia to 85% and end-tidal carbon dioxide to zero. The team lead performed a jaw thrust with laryngospasm notch pressure, hyperextension of the neck, and bag valve mask ventilation with appropriate seal. The end-tidal continued to read zero, with worsening oxygenation saturations to 45%, perioral cyanosis, and a downtrend in the heart rate. The team activated a code response and administered succinylcholine while continuing airway interventions. Bag valve mask ventilations were continued until the patient was maintaining airway and oxygen saturations. The mother returned to the room, and the team lead explained the events to her after which the patient awakened and was discharged home.

Debriefing
Debriefing with participants took place immediately after simulations. Allotted time for debriefing of each simulation was around 40 minutes. The materials in Appendix F were used to aid in discussion and were modeled after the PEARLS approach. Through PEARLS' three broad educational strategies (fostering learner self-assessment, facilitation of a focused discussion, and information provided via directive feedback) and its blended approach, debriefing was interactive and collaborative. Appendix H was provided to learners either prior to or after the simulation for further self-driven learning.

Assessment
Critical actions based on educational learning objectives were tracked throughout both simulations. Prompts were included in the simulations so that these critical actions would be completed. Both sedation simulations and debriefing were instructed and led by PEM providers with experience in simulation. Following sedation simulations, learners voluntarily provided feedback via a sedation simulation evaluation form (Appendix G). The form contained questions with answer options ranging from strongly disagree to strongly agree, as well as three open-ended questions meant to acquire qualitative feedback to improve the simulations and learners' overall experiences. Each institution's feedback allowed us to edit the scenarios and create improved learning tools for pediatric emergency medicine providers.

Results

Both sedation simulations were implemented at three institutions with 58 participants, including PEM fellows and pediatric and EM residents. All facilitators running the simulations were either current PEM fellows or faculty, with some faculty having additional simulation-based medical education training or experience. The simulations were geared towards both expanding learners' sedation knowledge base and reviewing prior knowledge. During the debrief in a safe environment, learners were able to give feedback on the cases and ask pertinent learning questions. At that time, learning objectives were reviewed, and any objectives that had required a prompt or had been missed were discussed

Copyright © 2024 Dupont et al. This is an open-access publication distributed under the terms of the Creative Commons Attribution license.

in detail. The educational PowerPoint was used to augment any missed learning objectives.

Immediately following the debrief sessions, 53 of the 58 participants (18 Phoenix Children's fellows, seven Medical College of Wisconsin fellows, 22 EM Vanderbilt residents, and six Vanderbilt fellows) filled out an evaluation that included five quantitative and three qualitative questions. The five quantitative questions were scored on a 5-point Likert scale (1 = *strongly disagree,* 2 = *disagree,* 3 = *neither agree or disagree,* 4 = *agree,* 5 = *strongly agree*). Learners rated the scenario as clinically relevant ($M = 4.37$) and effective at improving their comfort level in caring for critically ill patients ($M = 4.36$). Learners felt the debrief provided valuable learning ($M = 4.40$) and was a safe learning environment ($M = 4.50$). Qualitative feedback included the following: cases "increased comfort level with propofol as we primarily use ketamine," "great case," and "this was very helpful." Takeaway learning points included appropriate setup for airway compromise and hemodynamic instability when using propofol for sedation, review of dosing for medications, when to use succinylcholine, and the importance of a consent/assent discussion prior to sedation.

Discussion

These sedation simulation cases were written to improve providers' knowledge of and comfort level in providing procedural sedation in the ED. Ketamine is a common medication used in ED procedural sedations due to its analgesic and dissociative properties. Propofol, although generally less commonly used for pediatric procedural sedations, is another advantageous sedation medication. Given the high volume of procedural sedations performed in the PED, providers should be comfortable with obtaining consent/assent, preparing the room for sedation, dosing/redosing of ketamine and propofol, these medications' side effects, and the ability to manage side effects. Through the use of a high-fidelity mannequin in a realistic simulation scenario, providers can work on this skill set. This simulation was written so that instructors would have access to all potential resources needed to carry out the session in their own setting. Cues throughout are intended to help guide learners through the cases if they get stuck. A debriefing guide and an educational PowerPoint aid in postsimulation discussion and further learning.

Learner feedback included the need for further discussion on how to obtain proper sedation consent and assent from the patient's guardian and the patient, respectively. Future iterations of these simulations will feature a more in-depth discussion of obtaining consent during the debrief session and inclusion of this information on the educational PowerPoint. The need for further education around propofol was evident from the learner feedback as well. These simulations can be used for new trainees at the beginning of the academic year for institutions where ketamine and propofol are commonly used and as a review for trainees at institutions where propofol is not as commonly utilized.

Limitations include the inability to portray typical medication side effects, such as nystagmus and hypersalivation, with a high-fidelity mannequin. Secondly, we did not directly assess changes in comfort level and medical knowledge around pediatric procedural sedation medicine beyond the debriefing session. Given the high frequency with which PEM providers perform pediatric sedations, our hope is that providers can take the knowledge they absorb and immediately apply it clinically to maintain this skill set. The learning scenarios can be used either at the beginning of fellowship/residency to improve first-year fellow/resident comfort level with pediatric procedural sedation or throughout fellowship/residency as a means to review this material.

Pediatric ketamine and propofol sedations are commonly performed in PEDs. Providers should be comfortable obtaining consent, setting up for a procedural sedation, dosing and redosing sedatives, knowing side effects of these medications, and acting on these side effects. Although life-threatening side effects are rare, providers should be able to promptly act on them. These simulations are effective ways to practice high-acuity events so that, when they occur in real time, the provider is ready.

Appendices

A. Sedation Simulation Cases.docx
B. Sedation Simulation Patients.docx
C. Critical Actions Checklist.docx
D. Sedation Simulation Equipment.docx
E. Sedation Simulation X-Ray Images.docx
F. Sedation Simulation Debriefing Materials.docx
G. Sedation Simulation Evaluation.docx
H. Propofol and Ketamine.pptx

All appendices are peer reviewed as integral parts of the Original Publication.

Copyright © 2024 Dupont et al. This is an open-access publication distributed under the terms of the Creative Commons Attribution license.

Amanda Dupont, DO: Assistant Professor, Division of Pediatric Emergency Medicine, Department of Pediatrics, Medical College of Wisconsin; ORCID: https://orcid.org/0009-0003-7500-4989

Daisy Ciener, MD, MS: Assistant Professor, Division of Emergency Medicine, Department of Pediatrics, Vanderbilt University Medical Center

Cecilia Monteilh, MD: Assistant Professor, Division of Pediatric Emergency Medicine, Department of Pediatrics, Phoenix Children's

Anita Bharath, MD: Assistant Professor, Division of Pediatric Emergency Medicine, Department of Pediatrics, Phoenix Children's

Anita Thomas, MD, MPH: Associate Professor, Division of Pediatric Emergency Medicine, Department of Pediatrics, Seattle Children's

Katherine Wolpert, MD: Assistant Professor, Section of Pediatric Emergency Medicine, Department of Pediatrics, Seattle Children's

Jean Pearce, MD, MS: Assistant Professor, Division of Pediatric Emergency Medicine, Department of Pediatrics, Medical College of Wisconsin

Disclosures
None to report.

Funding/Support
None to report.

Prior Presentations
Dupont A, Ciener D, Monteilh C, et al. Multi-institutional pediatric sedation complication simulation. Poster presented at: PAS 2023 Pediatric Academic Societies Meeting; April 27-May 1, 2023; Washington, DC.

Ethical Approval
Reported as not applicable.

References

1. Alletag MJ, Auerbach MA, Baum CR. Ketamine, propofol, and ketofol use for pediatric sedation. *Pediatr Emerg Care*. 2012;28(12):1391-1395. https://doi.org/10.1097/PEC.0b013e318276fde2
2. Bellolio MF, Puls HA, Anderson JL, et al. Incidence of adverse events in paediatric procedural sedation in the emergency department: a systematic review and meta-analysis. *BMJ Open*. 2016;6(6):e011384. https://doi.org/10.1136/bmjopen-2016-011384
3. Chidambaran V, Costandi A, D'Mello A. Propofol: a review of its role in pediatric anesthesia and sedation. *CNS Drugs*. 2015; 29(7):543-563. https://doi.org/10.1007/s40263-015-0259-6
4. Miller KA, Andolfatto G, Miner JR, Burton JH, Krauss BS. Clinical practice guideline for emergency department procedural sedation with propofol: 2018 update. *Ann Emerg Med*. 2019;73(5):470-480. https://doi.org/10.1016/j.annemergmed.2018.12.012
5. Cravero JP, Beach ML, Blike GT, Gallagher SM, Hertzog JH. The incidence and nature of adverse events during pediatric sedation/anesthesia with propofol for procedures outside the operating room: a report from the Pediatric Sedation Research Consortium. *Anesth Analg*. 2009;108(3):795-804. https://doi.org/10.1213/ane.0b013e31818fc334
6. Deitch K, Chudnofsky CR, Dominici P. The utility of supplemental oxygen during emergency department procedural sedation with propofol: a randomized, controlled trial. *Ann Emerg Med*. 2008; 52(1):1-8. https://doi.org/10.1016/j.annemergmed.2007.11.040
7. Xue FS, Tong SY, Wang XL, Deng XM, An G. Study of the optimal duration of preoxygenation in children. *J Clin Anesth*. 1995;7(2): 93-96. https://doi.org/10.1016/0952-8180(94)00011-R
8. Statement on ASA Physical Status Classification System. American Society of Anesthesiologists. 2014. Updated December 13, 2020. Accessed January 5, 2024. https://www.asahq.org/standards-and-practice-parameters/statement-on-asa-physical-status-classification-system
9. Bajaj K, Meguerdichian M, Thoma B, Huang S, Eppich W, Cheng A. The PEARLS Healthcare Debriefing Tool. *Acad Med*. 2018; 93(2):336. https://doi.org/10.1097/ACM.0000000000002035

Received: July 7, 2023
Accepted: November 20, 2023
Published: February 13, 2024

Copyright © 2024 Dupont et al. This is an open-access publication distributed under the terms of the Creative Commons Attribution license.
